# Evolution of Integrated Causal Structures in Animats Exposed to Environments of Increasing Complexity

**DOI:** 10.1371/journal.pcbi.1003966

**Published:** 2014-12-18

**Authors:** Larissa Albantakis, Arend Hintze, Christof Koch, Christoph Adami, Giulio Tononi

**Affiliations:** 1 Department of Psychiatry, University of Wisconsin, Madison, Wisconsin, United States of America; 2 Department of Microbiology and Molecular Genetics, Michigan State University, East Lansing, Michigan, United States of America; 3 BEACON Center for the Study of Evolution in Action, Michigan State University, East Lansing, Michigan, United States of America; 4 Allen Institute for Brain Science, Seattle, Washington, United States of America; 5 Department of Physics and Astronomy, Michigan State University, East Lansing, Michigan, United States of America; University of Hertfordshire, United Kingdom

## Abstract

Natural selection favors the evolution of brains that can capture fitness-relevant features of the environment's causal structure. We investigated the evolution of small, adaptive logic-gate networks (“animats”) in task environments where falling blocks of different sizes have to be caught or avoided in a ‘Tetris-like’ game. Solving these tasks requires the integration of sensor inputs and memory. Evolved networks were evaluated using measures of information integration, including the number of evolved concepts and the total amount of integrated conceptual information. The results show that, over the course of the animats' adaptation, i) the number of concepts grows; ii) integrated conceptual information increases; iii) this increase depends on the complexity of the environment, especially on the requirement for sequential memory. These results suggest that the need to capture the causal structure of a rich environment, given limited sensors and internal mechanisms, is an important driving force for organisms to develop highly integrated networks (“brains”) with many concepts, leading to an increase in their internal complexity.

## Author Summary

The capacity to integrate information is a prominent feature of biological brains and has been related to cognitive flexibility as well as consciousness. To investigate how environment complexity affects the capacity for information integration, we simulated the evolution of artificial organisms (“animats”) controlled by small, adaptive neuron-like networks (“brains”). Task environments varied in difficulty due primarily to the requirements for internal memory. By applying measures of information integration, we show that, under constraints on the number of available internal elements, the animats evolved brains that were the more integrated the more internal memory was required to solve a given task. Thus, in complex environments with a premium on context-sensitivity and memory, integrated brain architectures have an evolutionary advantage over modular ones.

## Introduction

Many studies have sought to elucidate the role of information in evolution [1]–[4], its relation to fitness [5]–[7], and how information about the environment is acquired and inherited by an organism [8], [9]. Common to most current approaches to characterize and quantify information in biology is the notion that biological information has to be physically implemented and should be functional, meaning valuable to the organism and related to the environment [3], [4], [9]. There is also growing interest in how measures of information can shed light on the apparent growth in complexity during evolution [2], [10]–[12].

Artificial adaptive agents (“animats”) have proven useful for investigating how various information and complexity measures change during evolution [6], [7], [13]. Animats consist of small neural networks (“brains”), with sensors, hidden elements, and motors, which are evolved under selection based on task fitness. In recent work we used animats consisting of Hidden Markov elements evolving in a task environment that requires integrating current sensor inputs with memory. We showed that the animats' increasing fitness is associated with an increase in the capacity to integrate information [6], [7].

In this study, we extend these initial results in two ways. First, we evaluate the animats' capacity for integrated information using the comprehensive set of measures recently introduced in the context of integrated information theory (IIT 3.0, see Box 1 and [14], [15], for previous versions see [16] (“IIT 2.0”) and the original formulation for stationary systems [17], [18] (“IIT 1.0”)). Specifically, we ask whether adaptation to an environment leads to an increase in the number of evolved *concepts* and in the total amount of *integrated conceptual information* (*Φ^Max^, “Big Phi”*). Second, we compare how different task environments influence the evolution of animats and their capacity to integrate information depending on memory requirements and size of the sensory-motor interface. In this way, we aim to elucidate under which conditions integrated brains with high *Φ^Max^* become advantageous.

**Box 1. Integrated (Conceptual) Information**

Information and causation in physical systems are typically evaluated from the extrinsic perspective of an observer. By contrast, integrated information theory (IIT) [14]–[16], [18], [52] provides a theoretical framework to characterize the causal/informational structure of adaptive systems from the intrinsic perspective of the system itself (“differences that make a difference” to the system). A system is comprised of a set of mechanisms, where ‘‘mechanism’’ simply denotes anything having a causal role within the system (i.e., elements or sets of elements that (1) can assume different states depending on the rest of the system and (2) also influence the state of the rest of the system), for example, a neuron in the brain that can be “firing” or “not firing”, or a logic gate in a computer with “on” and “off” states. IIT invokes five postulates (stated explicitly in IIT 3.0 [15]) that lead to the definition of a fundamental quantity, *integrated information (“phi”)* that measures to what extent mechanisms (*φ)* and systems (sets of mechanisms) (*Φ*) are irreducible to their parts in causal/informational terms [14], [15]:

•**Existence:** From the intrinsic perspective of a system, only “differences that make a difference” [53] within the system matter. Therefore, the system's mechanisms must specify causes and effects *within* the system.
•**Composition:** The elements of a system can be structured, forming higher-order mechanisms.
•**Information:** The mechanisms of a system in a given state must specify the system's potential past and future states in a particular way. A conceptual structure is made up of the set of cause-effect repertoires specified by the system’s mechanisms (which past and future states of the system are possible given a mechanism and its current state).
•**Integration:** The conceptual structure specified by a system must be irreducible (*Φ*>0) to that specified by a partition of the system into non-interdependent sub-systems (minimum partition). Similarly, each mechanism must specify a cause-effect repertoire that is irreducible (*φ*>0) to that specified by its sub-mechanisms.
•**Exclusion:** Over a set of elements within a system, only one conceptual structure can be specified — the one that is maximally irreducible (*Φ^max^*). In that case the set of elements constitutes a *complex*. Exclusion avoids multiple causation: a mechanism that specifies a particular cause-effect repertoire within one complex cannot, in addition, specify an overlapping cause-effect repertoire as part of other, overlapping complexes. Otherwise, the difference that mechanism makes would be counted multiple times. Similarly, each mechanism can only specify one cause-effect repertoire, the one that is maximally irreducible (*φ* ^max^). In that case the mechanism constitutes a *concept*.

Information-theoretic approaches to assess the evolved complexity of (artificial) organisms are typically based on extrinsic correlational measures, either between the system's genome and its environment [8], [19] or between the system's sensors and motors [20] (sensory-motor information), or between successive system states [6], [7] (predictive information [21]). By contrast, IIT quantifies information from the intrinsic perspective of the system, based on the causal power of its internal mechanisms - the “differences that make a difference” within the system [14]–[16], [18], [22], [23]. In the animats employed here, a mechanism consists of one or more system elements that, at a given time, are in a particular state (on or off). A mechanism in a state specifies a *concept* if it meets the following conditions (see Methods for details). First, the mechanism must specify which past and future states of the system are possible and which are not (information). The particular way in which it does so constitutes its *cause-effect repertoire*, the probability distribution of past and future system states given the current state of the mechanism. Second, its cause-effect repertoire must be irreducible to that specified by sub-mechanisms (integration). Irreducibility of a mechanism is assessed by measuring its integrated information *φ* (“small phi”) - the distance between the cause-effect repertoire of the intact mechanism and that of its minimum partition (MIP), which renders the weakest connection of the mechanism causally ineffective. *φ* thus quantifies how much causal information is lost due to the MIP. A mechanism can specify only one cause-effect repertoire, the one that is *maximally* irreducible (exclusion, *φ^Max^*, see Methods). This constitutes its concept—what the mechanism in a state “does” for the system from the intrinsic perspective of the system itself.

The set of all concepts and associated *φ^Max^* values generated by a set of elements constitutes a *conceptual structure* (information, see Methods for details). As for individual concepts, the integration of a conceptual structure can be evaluated by measuring the distance *Φ* (“big phi”) between the conceptual structure of the intact set and that of its minimum (unidirectional) partition (see Methods). Within some animats, a set of elements may generate a maximally integrated conceptual structure (*Φ^Max^*), which constitutes a *main complex* (MC, exclusion). Other animats may not contain complexes (*Φ* = 0) because their brains are constituted of functionally segregated modules with feed-forward architecture (containing at most self-loops) [15]. In sum, *Φ* can be viewed as a measure of complexity, since only systems with many specialized, but integrated mechanisms have high *Φ*, whereas systems that have only a few different mechanisms and/or are very modular have low or no *Φ* [15], [16], [22].

From an engineering point of view, modular systems with segregated functions are much simpler to design and understand than integrated systems. However, simplicity of design is not an issue for evolution by natural selection. Instead, important factors are economy of elements/wiring [24], composition of functions [14], degeneracy (multiple ways to achieve the same function) [25], adaptability in the face of change [26], [27], integrated control [14], and robustness to failure [28]. These factors should favor the evolution of organisms with integrated brains in an environment that is complex, changing, and requires sensitivity to context [14], [25], [29]. Based on these considerations, we predict that measures of integrated information should increase with the complexity of the environment. Specifically, i) evolving animats should show an increase in the number of concepts; ii) integrated conceptual structures should become larger and more irreducible; iii) the increase in concepts and integrated conceptual structures should be related to the complexity of the environment and to the requirements for memory. Moreover, to the extent that IIT is correct in claiming that the capacity for information integration underlies consciousness [14], [15], [18], [23], finding an increase in animats' *Φ^Max^* values in complex environments would provide a plausible account of why and how consciousness evolved.

In what follows, we test and confirm these predictions by evolving animats solving perceptual categorization tasks [13], [30] in task environments that vary in the amount of sequential memory necessary to solve the task optimally. The results show that, given strict constraints on the number of elements in the animat's brain, integrated network architectures become advantageous over modular or feed-forward architectures when the environment was more complex. Moreover animats with restrictions on the number/fidelity of their sensors or motors evolved more concepts and larger integrated conceptual structures, in line with an increased reliance on memory.

## Results

In order to investigate the causal structure of a system from an evolutionary perspective, we simulated the adaptation of simple neural networks (“animats”) [6], [7], [13] in task environments of varying difficulty. For these animats, their concepts and integrated conceptual information *Φ* can be calculated rigorously across many generations (see Methods). This permits testing the following predictions about the evolution of (integrated) conceptual information during adaptation to specific environments:

1. The number of concepts and their summed *φ* ^max^ values should increase during adaptation, proportional to the amount of internal computation necessary to solve a task.
2. Given a limited number of hidden elements, integration should also increase during adaptation, particularly in tasks that require more memory.
3. Since the reliance on memory increases with the complexity of the environment relative to the sensor and motor capacities of the organism, the number of concepts and their integration should also increase during evolution under sensor or motor limitations.

### Animats and adaptation

**Figure 1:**
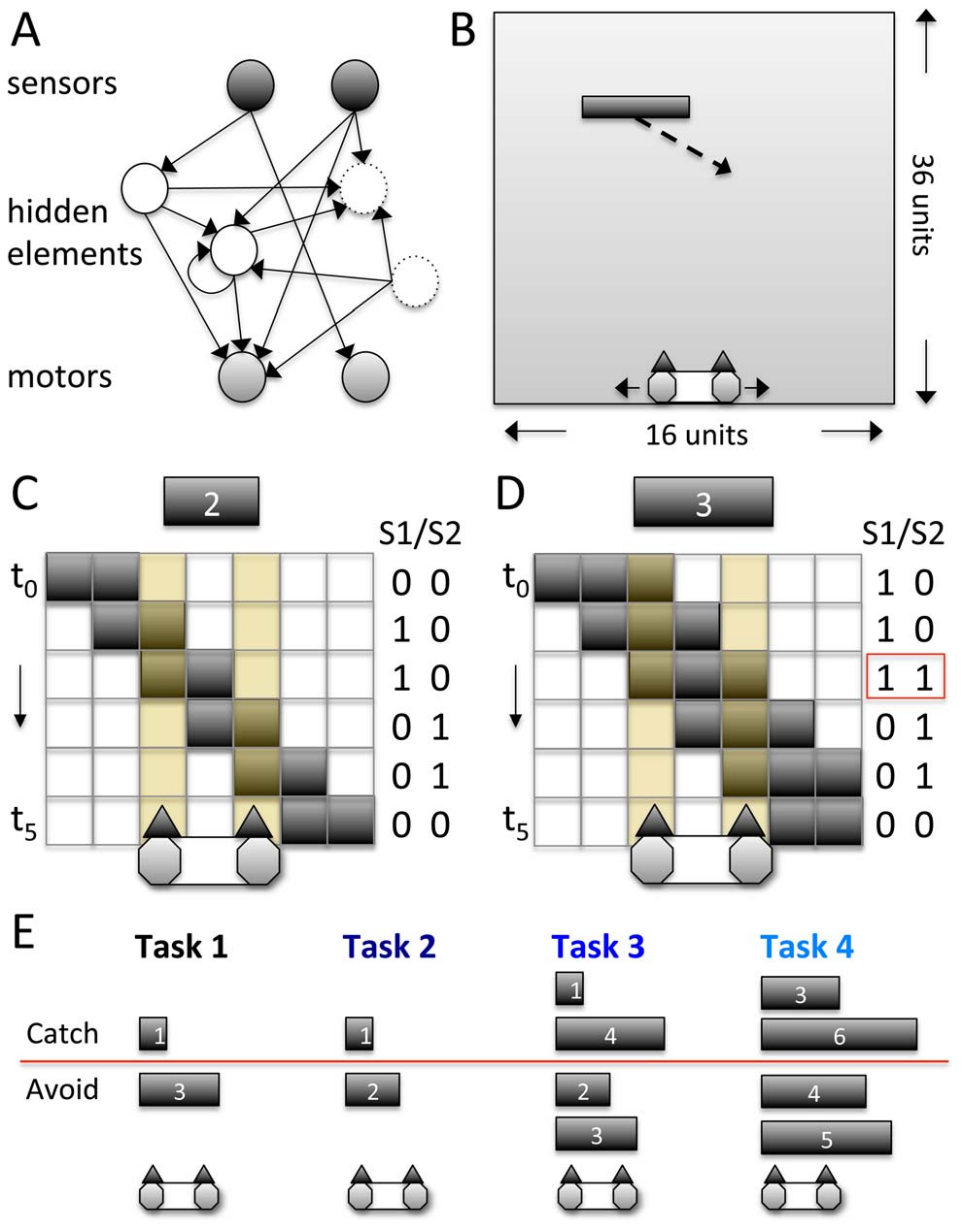
Animats and task environments. (A) Exemplar wiring diagram. Elements without causal role (unconnected elements, or hidden elements with inputs or outputs only) are dashed. Sensor elements can connect directly to motor elements. No feedback to the sensor elements or from the motor elements is allowed. (B) Schematic of animat in exemplar environment with periodic boundary conditions at the vertical walls (if a block e.g. moves out on the left it will reappear on the right). The animat has to distinguish the size of the downward moving blocks and either catch or avoid them. The animat is 3 units wide with a space of 1 unit between its sensors. Per trial, one block is positioned at one of 16 possible starting positions, 36 units above the animat. (C,D) Blocks continuously move either to the left or right, one unit per time step and also down at one unit per time step. If a block is positioned above a sensor element, the sensor switches on. (C) Pattern of sensor activation for a block of size 2 in case the animat is not moving. (D) The same for a block of size 3. Blocks with size ≥3 can activate both sensors at the same time. (E) Illustration of Task 1–4.

Each animat is equipped with a fixed number of sensors, hidden elements, and two motor outputs (to move either left or right, see Fig. 1). All elements are binary Markov variables, whose value is specified by deterministic logic gates. Each animat has a genome, which encodes the wiring diagram of the animat's brain and the logic functions of its elements. More precisely, each gene specifies a hidden Markov gate (HMG) and all HMGs together determine the brain's causal structure (see Methods and [6], [13]). The animats are allowed to evolve over 60,000 generations using a genetic algorithm, starting with an initial population of 100 animats without connections between brain elements (generation zero). To compose the next generation, the genetic algorithm selects a new sample of 100 animats based on an exponential measure of the animats' fitness (roulette wheel selection). The genome of each selected animat is mutated according to three probabilistic mutation mechanisms (point mutations, deletions, and duplications) [13]. The mutated genomes then determine the wiring diagrams and logic functions of the next animat generation, which are tested for fitness in the respective task environment. In sum, adaptation arises through mutation and selection driven by the animat's task performance.

Throughout this study, the animats' task environments are variants of “Active Categorical Perception” (ACP) tasks [13], [30], where moving blocks of different sizes have to be distinguished in a ‘Tetris-like’ game (Fig. 1B). Adaptation is measured as an increase in fitness, where fitness corresponds to the fraction of successfully caught or avoided blocks within a fixed number of trials (128 for each animat at each generation, with one falling block per trial). Blocks move sideways and down at 1 unit per time step either to the right or left starting from one of 16 possible initial positions. If a block moves out on the left it will reappear on the right and vice versa. A block is “caught” if the animat overlaps with at least one of its units when it reaches the bottom (after 36 time steps); otherwise the block is “avoided”. Each animat's size is 3 units, with a space of 1 unit between the two sensors (a “blind spot”). Therefore, only blocks of size ≥3 can activate both sensors at the same time (Fig. 1C,D). Note that the sensors of the animat convey limited information about the environment and only at a single time step, yet solving ACP tasks successfully requires integration of sensor inputs over multiple time steps. Hence, information about past sensor states (memory) has to be stored through the states of internal elements.

At the end of each evolutionary run at generation 60,000, the line of descent (LOD) of one animat is traced back through all generations. Every 512 generations along the LOD, a transition probability matrix (TPM) is generated for all possible states of the animat's brain, which captures how the brain transitions from one state to another. From these TPMs, concepts and integrated conceptual information *Φ* can be calculated across the LOD. We averaged the causal measures for a particular generation in one LOD across all network states experienced by the animat during the 128 test trials, weighted by their probability of occurrence. For each task condition, 50 independent LODs were obtained, each from a different evolutionary run.

### IIT predictions on Tasks 1–4

To investigate how the number of concepts and their integration depends on the causal structure of the task environment, we tested the animats in four tasks (Task 1–4) with different block categories and strategic requirements (Fig. 1E). Given the periodic boundary conditions and the fact that the animats can actively explore their environment, predicting the evolutionary difficulty of an ACP design is not straightforward. Nevertheless, if solving a task requires more memory of input sequences, the number of concepts developed by the animats should increase. Since the number of evolvable hidden elements is limited to four, the number of time steps that can be combined without feed-back between elements and thus *Φ* = 0 (see Methods and [15]) is limited, too. Higher memory requirements should thus bias the animats towards developing brains with more integrated conceptual structures with larger main complexes and higher *Φ*.

As a first simple task environment (Task 1), the animats have to catch blocks of size 1 and avoid blocks of size 3. In Task 1, the two block conditions can in principle be distinguished based on a momentary sensor state (S_1_S_2_ = 11, see Fig. 1C,D). Categorization can thus be achieved in a modular manner (e.g., “if S_1_S_2_ = 11 avoid, else follow”). However, memory is still required to identify the direction of the moving blocks, since sensor information of at least two time steps must be combined to infer movement direction. Task 1 will serve as the comparison environment in the following sections.

In Task 2, the blocks to be avoided are smaller (2 units). Consequently, the two block categories cannot be distinguished based on a single sensor state, since neither block can activate both sensors at the same time. Here, memory is required for both categorization of block size and direction.

In Task 3, four instead of just two different block sizes have to be distinguished. The blocks to be caught (size 1 and 4) and avoided (size 2 and 3) cannot be distinguished based on a single threshold (e.g. “≥3”), nor based on a single sensor state. Adaptation to Task 3 is thus expected to be more difficult. However, sensor state S_1_S_2_ = 11 allows distinguishing blocks of size 1 and 2 from blocks of size 3 and 4. Whether to catch or avoid a block can then be decided based on a memory of one time step, just as in Task 2. Note also that in Task 3 at least 75% fitness can be achieved with the same categorization strategy as in Task 2 (“≥2”). Therefore, more concepts than in Task 2 are expected only for fitness levels>75%.

Finally, in Task 4, four blocks of sizes ≥3 have to be distinguished. To successfully catch blocks of size 3 and 6 and avoid blocks of size 4 and 5 the animats have to combine memory of at least 3 time steps.

In sum, the evolutionary pressure to develop brains with integrated concepts should be lowest for Task 1, intermediate for Task 2/3, and highest for Task 4, in line with the requirements of sequential memory in Task 1–4. According to IIT, both the average number of concepts and their integration (*Φ^Max^*) should therefore be highest in Task 4 and lowest in Task 1.

### Comparing number of concepts and *Φ* in Task 1–4

**Figure 2:**
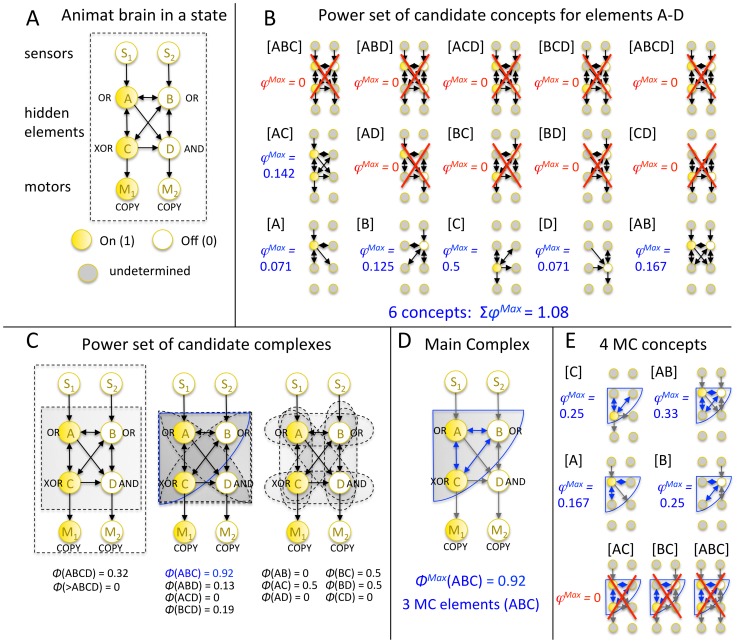
Assessing the causal structure of an animat in a state. (A) A hypothetical animat brain comprised of a logic-gate network with 2 sensors (S_1_S_2_), 4 hidden elements (ABCD), and 2 motors (M_1_M_2_) is analyzed for illustration in state 00-1010-10. (B) First, the power set of all candidate concepts in the entire animat brain is evaluated. Note that the sensors and motors cannot give rise to concepts or be part of higher order concepts since – by design - they either lack causes or effects (i.e., inputs or outputs) within the system. Each animat brain can thus maximally have 2^4^−1 = 15 concepts (the power-set of the 4 hidden elements, excluding the empty set). “Small phi” φ measures how irreducible a mechanism's cause-effect repertoire is over a particular set of inputs and outputs. φ^Max^ is the integrated information of the most irreducible cause-effect repertoire of the mechanism. The number of concepts and Σφ^Max^ are measures of all the brain's causal relations and their strength, both modular or feed-forward and integrated. Here, 6 concepts exist, 4 elementary concepts ([A], [B], [C], [D]) and 2 higher order concepts ([AB], [AC]). All other higher order mechanisms are reducible (φ^Max^ = 0). (C) Second, Φ (“big phi”) is evaluated for all subsets of the system (candidate complexes). Φ measures how integrated a set of elements is. It quantifies how much the concepts of the set of elements change under a unidirectional partition between elements (for example, “noising” the connections from A to the rest of the system, leaving the connections to A from the system intact, see Methods). During the analysis, elements outside of the candidate complex are taken as fixed background conditions and remain unperturbed. Note that all subsets that contain either a sensor or a motor have Φ = 0, because elements that are connected to the rest of the system in a feed-forward manner cannot be part of an integrated system (see Methods). An animat's main complex can thus contain at most the 4 hidden elements. (D) Of all subsets of elements, in this particular system state, ABC is maximally integrated (Φ^Max^ = 0.92) and thus forms the main complex (MC). Gray arrows denote fixed background conditions, blue arrows denote functional connections within the MC. (E) Out of the power-set of ABC (maximally 2^3^−1 = 7 possible concepts), the MC specifies 4 irreducible concepts. The number of elements of the main complex, the number of MC concepts, and Φ^Max^ measure different aspects of how integrated the animat's brain is. For each animat at a particular generation, the analysis is performed for every state of the animat's brain, while the animat is performing its particular task. The state-dependent values are then averaged, weighted by the probability of occurrence of each state over 128 trials of different blocks falling.

Throughout the following analysis, the animats are evaluated in two ways: first, all concepts and the sum of their *φ^Max^* values are calculated for the animat's brain as a whole, including the sensors, motors, and all hidden elements. These measures quantify *all* causal relations (“IF-THEN”) in the animat's brain. Second, the main complex (MC) within the animat's brain is identified and the number of elements that form the MC (“MC elements”), the number of concepts in the MC (“MC concepts”), and its *Φ^Max^* value are calculated according to IIT 3.0 [15]. These measures quantify the amount of integration in the animat's brain. In this way, increases in fitness that rely on integrated structures can be distinguished from those that can be achieved with modular networks with feed-forward architecture (containing at most self-loops). Fig. 2 illustrates all the causal measures of a potential animat brain in one particular state. The maximal possible number of concepts specified by an animat's brain is 15 (2^4^−1, the power-set of all hidden elements excluding the empty set, see Fig. 2B). An animat's main complex can, at most, comprise the 4 hidden elements. Determining upper bounds for *Σφ^Max^* and *Φ^Max^* is not straightforward (see S1 Text). In the present set of simulations, the overall highest observed values for an animat in a particular state were *Σφ^Max^* = 3.11 and *Φ^Max^*  = 4.125. Note that all the above measures are state-dependent [15]. At a particular generation, these measures are evaluated for every brain state experienced by the animat during the test trials. The resulting state-dependent values are then averaged, weighted by the probability of occurrence of each brain state.

**Figure 3:**
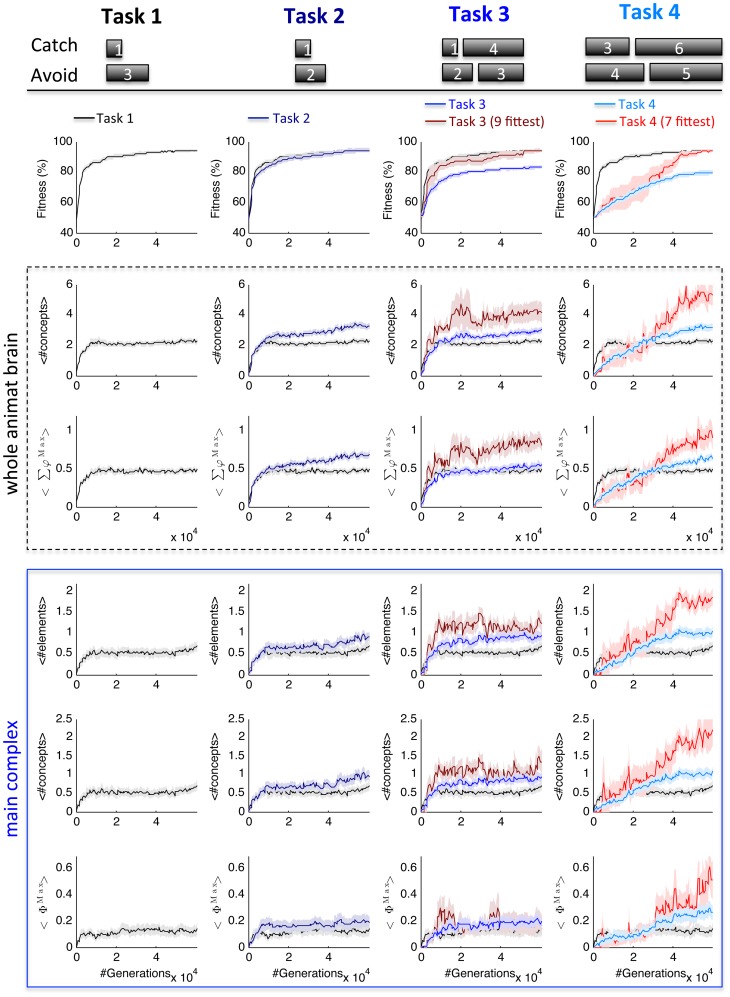
Comparison of concepts and integration across different task environments. Fitness, the average number of concepts and their <Σφ^Max^> values in the whole animat brain, and the average number of MC elements, MC concepts, and <Φ^Max^> of Tasks 1–4 were measured for 50 independent LODs. All animats were evolved for 60,000 generations. Shaded areas indicate SEM. The block sizes that had to be caught or avoided for the respective tasks are indicated at the top. For comparison, Task 1 is shown in black in every column. Task 1: The average fitness increases rapidly at first (to ∼82% in 5000 generations), followed by a slower increase to 93% at generation 59,904. The mean number of concepts specified by all elements comprising the animats' brains and their mean <Σφ^Max^> increased during adaptation. The animats also developed main complexes with increasing mean number of MC elements, MC concepts, and mean <Φ^Max^> value, albeit with higher variability between the different LODs. Task 2: In contrast to Task 1, the two different block sizes in Task 2 could not be distinguished based on a momentary sensor state since both blocks are <3. The difficulty of Task 2 is similar to Task 1—the same average level of fitness is reached. Nevertheless, the animats developed more concepts and higher <Σφ^Max^>. Also the average MC measures show higher values in Task 2 for generations>40,000, but to a lesser degree (see text). Task 3/4: The animats had to distinguish four different block sizes. Task 3 and 4 were thus more difficult: the average fitness reached after 60,000 generations is lower (83% and 80%) than in Task 1 and 2 (93% and 94%). The average measures across all 50 LODs are shown in blue (columns 3 and 4). To compare the causal measures independent of differences in fitness, we also analyzed the subsets of LODs with highest final fitness that on average best matched that of Task 1 (shown in red, columns 3 and 4, see Methods). As expected, in Task 3, only the subset that reached high fitness evolved more concepts than Task 1. Yet, even considering all 50 LODs, MC measures showed higher values, similar to those of Task 2. In Task 4 all causal measures reached higher values than in Task 1, particularly for the subset of LODs with high fitness.

Fig. 3 shows the evolution of all causal measures during adaptation over 60,000 generations in all four task conditions. For each task condition, 50 independent LODs are assessed every 512 generations. In Table 1, the average Spearman rank correlation coefficients across all 50 LODs are listed for all measures and tasks. As previously observed in a different kind of task environment [7], trial-by-trial correlation coefficients with fitness were rather broadly distributed (see histograms in S1 Fig.). While the causal measures are interrelated to some extent and the MC measures in particular tend to correlate, dissociations among them occur for individual LODs (see S2 Fig. for examples).

**Table 1:** Average Spearman rank correlation coefficients <R> across all 50 LODs between all applied measures and fitness.

		<#concepts>	<Σφ^Max^>	<#MC elements>	<#MC concepts>	<Φ^Max^>
***Task 1***	**<R>**	0.38	0.28	0.13	0.14	0.11
	**SEM**	0.05	0.05	0.05	0.05	0.05
***Task 2***	**<R>**	0.55	0.48	0.22	0.22	0.21
	**SEM**	0.04	0.04	0.05	0.05	0.05
***Task 3***	**<R>**	0.47	0.39	0.30	0.28	0.27
	**SEM**	0.04	0.04	0.05	0.05	0.05
***Task 4***	**<R>**	0.71	0.63	0.47	0.48	0.50
	**SEM**	0.04	0.03	0.06	0.06	0.06

Since the average number of concepts and their *Σ*φ^Max^ values capture both modular and integrated causal relations in the animat's brain as a whole, they correlated more strongly with fitness than the average number of MC elements, MC concepts and <*Φ* ^Max^>, which nevertheless increased with adaptation. See S1 Fig. for complementary histograms of the correlation coefficients of all individual LODs.

**Figure 4:**
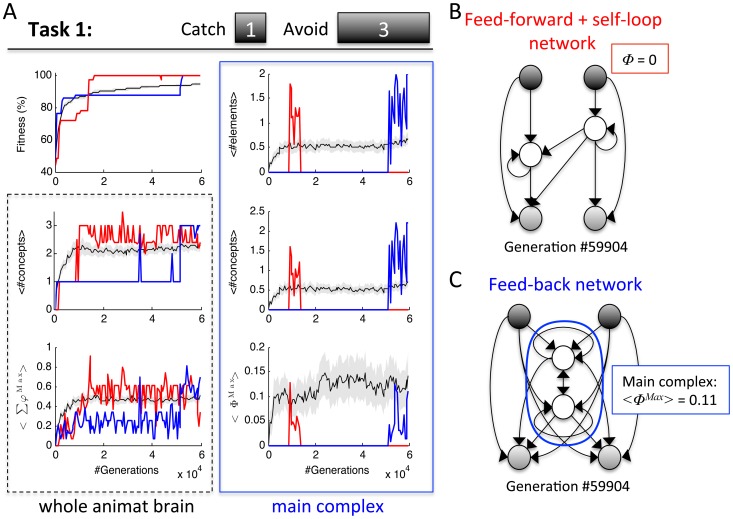
Task 1 can be solved in a modular and integrated manner. (A) Evolution of fitness, concepts, and integration across 60,000 generation. Two individual LODs are shown for two evolutionary histories in which the animats reached perfect fitness: in one history (blue) the animats developed an integrated main complex (<Φ^Max^>  = 0.10 at generation 59,904); in the other history (red), the animats developed a feed-forward structure with two self-loops (Φ^Max^ = 0 at generation 59,904). The red LOD, moreover, is a good example for dissociation between the MC measures and the number of concepts and their <Σφ^Max^> in the whole animat brain (generation 13,824). As in Fig. 3, the average across 50 animats (LODs) is shown in black, SEM in gray. (B) Wiring diagram at generation 59,904 for the red LOD that developed a modular network. (C) Wiring diagram at generation 59,904 for the blue LOD that developed an integrated network.

*Task 1 (* *Fig. 3*, *1st column):* At generation 59,904 the average fitness across all 50 LODs was 94.2±0.7% (mean ± SEM); in 13 out of 50 evolutionary lines the animats reached perfect fitness. On average, all causal measures were found to increase during the initial steep rise in fitness. The number of concepts and their *Σφ^Max^* values measured in the whole animat brain showed significant positive correlation with fitness (p<0.05) in 34/50 LODs. MC measures only correlated positively with fitness in 12/50 LODs (S1 Fig.), reflecting the fact that both modular (functionally segregated) and integrated concepts can lead to an increase in fitness. In other words, not every increase in fitness requires an increase in integration. In the case of Task 1, perfect categorization can be achieved with a purely modular (no MC, *Φ^Max^* = 0, 7/13 animats) as well as with an integrated network (*Φ^Max^*>0, 6/13 animats, see below, Fig. 4).

*Task 2 (* *Fig. 3* *, 2^nd^ column)*: In terms of adaptation, Task 2 was as difficult as Task 1 since the same level of fitness was reached (94.0±1.2%). Perfect fitness was achieved in 22/50 LODs. 16 out of these 22 animats developed integrated brains. Compared to Task 1 (black), with increasing fitness in later generations the animats developed brains with more concepts and higher *Σφ^Max^* values in Task 2 (U_98_ = 695.5/749.0, Z = −3.844/−3.454, p = 0.000/0.001 respectively for #concepts/*Σφ^Max^* averaged across the last 3,000 generations). MC measures in Task 2 increased more subtly, but reached higher values than in Task 1 (U_98_ = 985/966/922, Z = −1.899/−2.035/−2.350, p = 0.058/0.042/0.019 respectively for #MC elements/#MC concepts/*Φ^Max^*). The number of LODs with significant positive correlation with fitness (p<0.05) was also higher than in Task 1 for number of concepts and *Σφ^Max^* (42/50) and MC measures (24/50).

**Figure 5:**
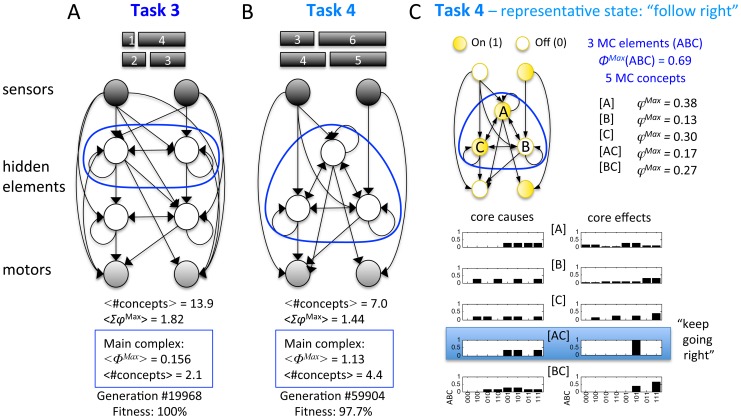
Wiring diagrams of fittest animats in Task 3 and 4. (A) In Task 3, perfect fitness was achieved temporarily in one LOD only. The fittest evolved animat had 4 hidden elements; two of them form a main complex. <#concept>, <Σφ^Max^>, and <Φ^Max^> are averages across all states experienced by the animat while performing the task weighted by probability of occurrence of each state. Note that this perfect Task 3 animat developed a very large overall number of concepts and high <Σφ^Max^>, while its MC values are comparable to Task 1/2 animats with perfect fitness and integrated MCs (Fig. 4C). (B) In Task 4, the fittest animat achieved a fitness level of 97.7%. The animat's hidden elements formed a main complex in all experienced states. Shown is the largest MC consisting of all 3 evolved hidden elements. In some states, however, the MC was comprised of only two hidden elements. Note that the average number of MC concepts was higher than the maximal number of 3 MC elements, which means that the main complex gave rise to higher order concepts. (C) Conceptual structure of the animat shown in B, for one representative state. This state is active, whenever the animat follows a block to the right (right sensor and motor are on). The animat's conceptual structure comprises 5 MC concepts, the elementary concepts A, B, and C and the 2^nd^ order concepts AC and BC. The cause-effect repertoires of the MC concepts are always about the elements within the main complex (ABC). Nevertheless, some concepts allow for interpretation from an extrinsic point of view: the higher order concept AC = 11, for example, specifies that coming from any of three possible past states (ABC = 001, 101, or 111), the next state of ABC will again be 101. Since this state is associated with switching the right motor on, the concept AC can be interpreted as “keep going right”. Interestingly, in the state associated with “follow left” (not shown), a corresponding 2^nd^ order concept AB = 11 exists, which can be interpreted as “keep going left”.

*Task 3 (* *Fig. 3*, *3^rd^ column)*: The average fitness reached at generation 59,904 was 82.9±1.0%. Perfect fitness was achieved only temporarily in one LOD (with final fitness 98.4%). The average number of concepts and *Φ^Max^* evolved to higher values in Task 3 compared to Task 1 (black) (U_98_ = 854/899, Z = −2.746/−2.530, p = 0.006/0.011 for #concepts/*Φ^Max^*), while *Σφ^Max^* and the number of MC concepts and MC elements stayed comparable to those of Task 1. To compare the different tasks without confounding effects due to differences in fitness, a subset of LODs with high final fitness was chosen out of the 50 LODs of Task 3, so that the average fitness across the last 5,000 generations matched that of Task 1 (9 fittest LODs, shown in dark red). When compared at the same level of fitness, all causal measures evolved to significantly higher values, except for the number of MC concepts, which still almost reached significance p<0.05 (U_57_ = 77.5/71.0/136/141/112, Z = −3.143/−3.247/−1.999/−1.886/−2.538, p = 0.002/0.001/0.046/0.059/0.011 respectively for #concepts/*Σφ^Max^*/#MC elements/#MC concepts/*Φ^Max^*). As predicted, the evolutionary pressure for concepts and integration in Task 3 appeared to be comparable to that of Task 2. Accordingly, trial-by-trial positive correlation with fitness in Task 3 was also similar to Task 2: number of concepts and *Σφ^Max^* correlated significantly with fitness in 39/50 LODs; MC measures correlated significantly with fitness in 24/50 LODs. At comparable average fitness levels, the fact that four instead of just two blocks had to be distinguished only led to a marginal increase in the number of concepts and their integration, since the requirement for sequential memory remained comparable between Task 2 and 3. Solving the more difficult Task 3 perfectly, however, might still require significantly more overall concepts and higher *Σφ^Max^* values than Task 2, since the perfect solution requires distinguishing the 4 different block sizes under every initial condition (see below, Fig. 5A).

*Task 4 (* *Fig. 3*, *4^th^ column)*: As expected, Task 4 was the most difficult task in terms of adaptation with an average final fitness of 79.5±1.4% at generation 59,904. The highest overall fitness reached across all 50 LODs was 97.7% (125/128 correct trials) in one LOD. Despite the lower fitness reached, the average number of concepts, *Σφ^Max^*, and *Φ^Max^* were significantly higher in Task 4 than in Task 1 (U_98_ = 813/862/850, Z = −3.034/−2.675/−2.879, p = 0.002/0.007/0.004 for #concepts/*Σφ^Max^*/*Φ^Max^*). More evolutionary pressure for sequential memory thus led to causal structures with a higher number of concepts and more integration. This became even more evident when comparing a subset of LODs of Task 4 with equivalent average fitness (fittest 7 LODs) to Task 1 (U_55_ = 28.5/47/53/52/33, Z = −3.604/−3.112/−3.159/−3.185/−3.677, p = 0.000/0.002/0.002/0.001/0.000 for #concepts/*Σφ^Max^/*#MC elements/#MC concepts/*Φ^Max^*). In this subset, the evolved *Φ^Max^* of Task 4 was significantly higher than in any of the other tasks (U_55/55/14_ = 33/61/11, Z = −3.677/−2.792/−2.170 compared to Task 1/2/3). Also most other causal measures were significantly higher than in Task 2 (U_55_ = 62.5/103/66/74, Z = −2.740/−1.751/−2.670/−2.474, p = 0.006/0.080/0.008/0.013 for #concepts/*Σφ^Max^/*#MC elements/#MC concepts). Moreover, the number of LODs positively correlated with fitness was highest in Task 4: in 48/50 LODs the number of concepts and *Σφ^Max^* correlated significantly with fitness, and the MC measures correlated significantly with fitness in 33/50 LODs.

Taken together, comparing the causal measures across different task environments confirmed the predictions of IIT: the number of concepts that evolved during adaptation and their integration was higher in those tasks that required more memory and that could not be solved based on momentary sensor inputs – lowest for Task 1, intermediate for Task 2/3, and highest in Task 4.

### Features of network structures evolved in Task 1–4

Given the restrictions imposed on the animats' brains (binary elements and at most 4 hidden elements), evolutionary selection based on task fitness provides a driving force for more concepts and their integration proportional to the amount of memory necessary to solve the tasks. This can be illustrated by considering the evolved network structures with high fitness in Task 1–4.

In Task 1 the maximum fitness reached with just one hidden element was 92.2% (118/128 correct trials). Yet, perfect fitness in Task 1 can be achieved in both a modular and integrated manner, i.e., with network structures with either *Φ^Max^* = 0 or *Φ^Max^*>0 (Fig. 4). Out of the 13 LODs in which animats reached perfect fitness, 7 developed modular networks. An example LOD is shown in red in Fig. 4A. In this example, an initial increase in fitness at generation 9,216 was accompanied by an increase in integration. Subsequently, however, the animat's brain turned modular again at generation 13,824 (*Φ* = 0), which in this case led to a jump in fitness. The evolved network structure is shown in Fig. 4B for generation 59,904. The two hidden elements have memory in the form of self-loops, which however does not count as integration (*Φ* = 0, since single units cannot form a MC because they cannot be partitioned). In all of the 7 independent LODs that led to perfect fitness and a modular brain, the final generation of animats had evolved the same functional wiring diagram and similar logic functions with only 2 types of behavior (low degeneracy).

In the remaining 6 LODs in which animats achieved perfect fitness, they evolved an integrated main complex with feedback between elements. An example LOD is shown in blue in Fig. 4A. The initial increase in fitness of that LOD to 87.5% was achieved without a main complex (*Φ^Max^* = 0) and just one concept in the whole animat brain (generation 8,704-51,200). The rapid increase to 100% fitness at generation 52,224, however, was preceded by the formation of a main complex (*Φ^Max^*>0) and thus integration of concepts at generation 51,712. In Fig. 4C the final evolved wiring diagram at generation 59,904 is shown. This network structure is predominant among the evolved animats that reached perfect fitness in an integrated manner (5 out of 6). Despite this “anatomical” uniformity, the evolved logic functions, and thus the evolved behavior of the animats in the final generation, differed for all 6 LODs (high degeneracy). Analyzing all animats with perfect fitness across all generations and LODs, the animats with *Φ^Max^*>0 showed 341 different TPMs, leading to 332 different behavioral patterns, which were implemented by 15 different wiring diagrams. By contrast, animats with *Φ^Max^* = 0 had only 60 different TPMs, leading to 44 different behavioral patterns, which were implemented by 11 different wiring diagrams. Moreover, once a solution (perfect fitness) with *Φ^Max^* = 0 was encountered, subsequent descendants with *Φ^Ma^*>0 networks (and vice versa) were rather rare and the variability of TPMs within one LOD was lower for modular networks with *Φ^Max^* = 0 than for integrated networks (see Fig. 4A and S3 Fig.). This indicates that, while solutions with *Φ^Max^* = 0 were encountered with about equal probability to *Φ^Max^*>0 solutions across 50 independent LODs, within a LOD neutral mutations without decrease in fitness happen more frequently given integrated networks. Recurrent networks with *Φ*>0 are thus more flexible, in the sense that there are other solutions close by on the fitness landscape, which can be reached through neutral mutations. Taken together, perfect adaptation to Task 1 seems to require at least 2 hidden elements, but could be achieved in a recurrent/integrated and feed-forward/modular manner with about equal likelihood. However, animats with perfect fitness and *Φ^Max^*>0 showed higher degeneracy and variability in structure and behavior (see also S3 Fig.).

In Task 2 the maximum fitness reached with just one hidden unit was only 75% (96/128 correct trials) compared to 92.2% in Task 1. The fact that the two categories of blocks in Task 2 have to be distinguished based on memory without the possibility to rely on momentary evidence thus appears to increase the evolutionary pressure to develop more hidden elements. Nevertheless, in Task 2 as well, perfect fitness was achieved with both modular (*Φ* = 0) and integrated networks (*Φ*>0). However, out of the 22 independent LODs with perfect fitness only 6 showed no integration of concepts (*Φ* = 0) at generation 59,504, with the same wiring diagram as shown in Fig. 4C (Task 1) in 5 out of 6 cases. Of the remaining 16 animats with perfect fitness and integrated MCs, half evolved 2 hidden elements and half 3, with 9 different types of wiring diagrams and even higher degeneracy in their evolved logic functions and behavior. This corroborates the fact that evolutionary pressure for more concepts and integration is higher in Task 2 than in Task 1. As in Task 1, degeneracy and variability in network structure and behavior in Task 2 was higher for animats with integrated brains: taking all animats with perfect fitness across all generations and LODs into account, the animats with *Φ^Max^*>0 showed 920 different TPMs, leading to 407 different behavioral patterns, implemented by 34 different wiring diagrams, compared to only 235 different TPMs, with 85 different behavioral patterns, implemented by 30 different wiring diagrams for animats with *Φ^Max^* = 0.

Although Task 3 and 4 were more difficult, the maximal fitness that was reached with just one hidden element in these tasks was similar to that of Task 2: 78.1% (100/128) in Task 3 and 77.3% (99/128 correct trials) in Task 4. However, even with 2 hidden elements, the highest overall fitness reached was only 96.9% (124/128 correct trials) in Task 3 and 93.8% (120/128 correct trials) in Task 4. While in Task 3 the highest fitness achieved with a modular network without an integrated main complex (*Φ* = 0) was 96.1%, in Task 4 it was only 89.8%. The wiring diagrams of the fittest animats of both tasks are displayed in Fig. 5. In both cases, the animats developed brains with more than two hidden elements and an integrated main complex. Notably, the fittest animat in Task 4 evolved a main complex that was strongly integrated with <*Φ^Max^*>  = 1.13 and had many higher order concepts. Fig. 5C shows the conceptual structure of the fittest animat of Task 4 for one representative state. While the MC concepts are always about the elements in the main complex, some may be interpreted from the extrinsic perspective, such as the concept AC = 11, which here could mean “keep going right”. Which concepts exist at a given time depends on the state of the system. In this way, evolved concepts can correlate with and indirectly refer to specific states/events of the environment. A detailed interpretation of the extrinsic and intrinsic meaning of the animats' MC concepts is, however, beyond the scope of this study. Although it cannot be excluded that Task 4 is in principle solvable with 4 hidden elements connected in a non-integrated manner (*Φ* = 0), these results suggest that evolution strongly prefers integrated brains in Task 4.

In summary, under the constraints of maximally 4 binary, hidden elements, the fittest animats evolved in Task 1 developed modular and integrated wiring diagrams with similar likelihood. With higher memory requirements evolution increasingly selected for integrated networks with *Φ^Max^*>0. In Task 4, all animats with>90% fitness (8 LODs) developed an integrated main complex.

### Reduced sensor/motor capacity requires more concepts and higher integration in the same task

Task difficulty and the amount of sequential memory necessary to solve a task depend not only on the environment, but also on the sensor and motor capacities of the animats themselves. Solving the same task with fewer (or worse) sensors and motors requires increased reliance on memory. Consequently, the animats' evolved number of concepts and their integration should increase if the animats' sensor and motor capacities are restricted during adaptation.

**Figure 6:**
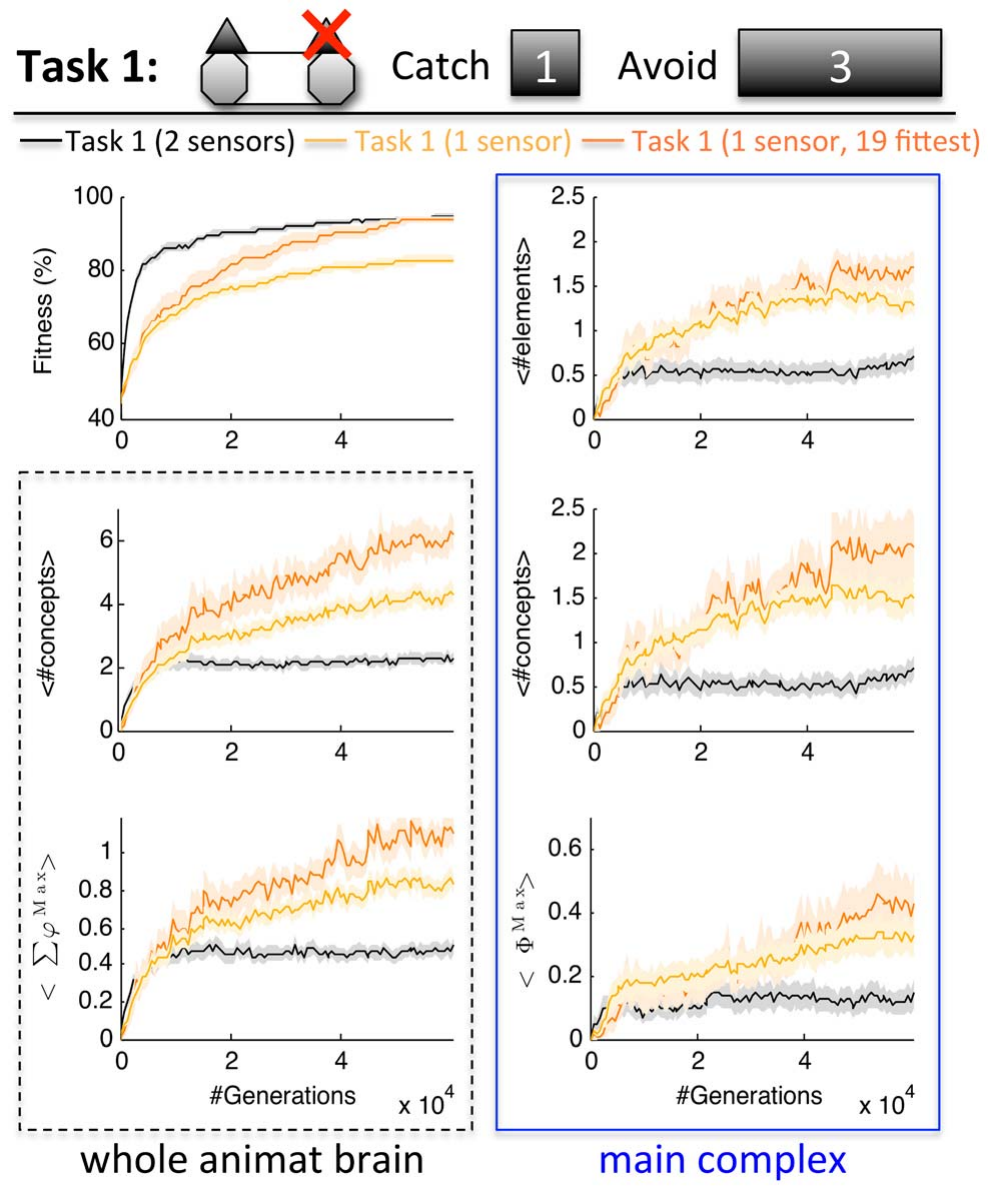
Concepts and integration in Task 1 with just one functioning sensor. Given only one sensor, Task 1 requires sequential memory for block and direction categorization. As a consequence the animats developed brains with more concepts and main complexes with more elements, concepts, and higher Φ^Max^ than with two sensors. The number of evolved concepts and their integration in Task 1 with one sensor was comparable to Task 4, the task that requires most sequential memory (Fig. 3, 4^th^ column).

To test this hypothesis, 50 additional LODs were evolved in the environment of Task 1 with one of the animats' sensors disabled (set to 0 at each time step and thus rendered useless). As explained above, with two functional sensors the two blocks in Task 1 can be categorized based on momentary sensory data alone (Fig. 2C,D). As a result, Task 1 could be solved equally well with a modular and integrated brain network (Fig. 4). Given only a single sensor, however, the task becomes more complex and requires memory of input sequences for block categorization. Fig. 6 shows the results obtained from the animats with only one sensor compared to Task 1 with two sensors (in black).

The average fitness reached with just one sensor was 82.8±1.4%. Nevertheless, in 4/50 LODs the animats reached 98.4% fitness (126/128 correct trials). As predicted, the animats evolved brains with more concepts, higher *Σφ^Max^*, and more integration than those with two sensors at their disposal (U_98_ = 510.5/514/746.5/749.5/728.5, Z = −5.116/−5.074/−3.591/−3.566/−3.716, p = 0.000, respectively for #concepts/*Σφ^Max^/*#MC elements/#MC concepts/*Φ^Max^*). Also the number of LODs that correlated positively with fitness was higher with only one sensor: number of concepts and *Σφ^Max^* correlated significantly in 46/50 LODs and MC measures in 36/50 LODs (compared to only 34/50 and 12/50, respectively, with two sensors). The increase in concepts and integration due to restricted sensors is even more apparent in the subset of 19 fittest LODs with the same average final fitness as in Task 1 with two sensors (Fig. 6, dark orange).

In terms of network structure, with just one sensor, the maximal fitness achieved with one hidden element was only 67.2% (compared to 92.2% with two sensors) and 95.3% with two hidden elements (100% with two sensors). In three out of the four fittest LODs (98.4% fitness), the animats evolved brains with an integrated main complex (*Φ*>0). Overall, the results obtained in Task 1 with one sensor are comparable to those of Task 4, the task with the largest block sizes, which requires most sequential memory (Fig. 3, 4^th^ column).

**Figure 7:**
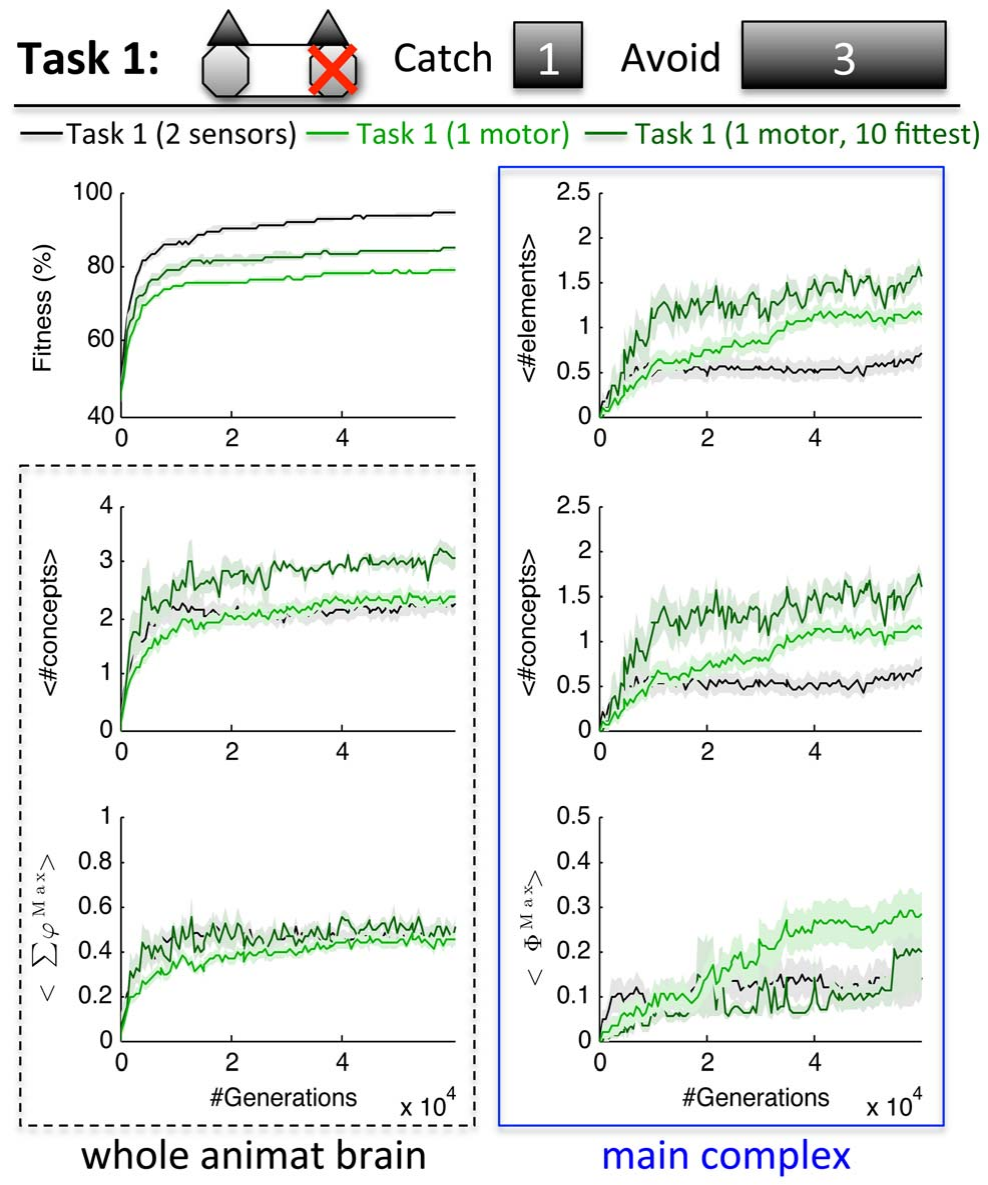
Concepts and integration in Task 1 with just one functioning motor. Given only one motor, Task 1 requires sequential control of the motor element. As a consequence the animats developed main complexes with more elements, concepts, and higher Φ^Max^ than with two motors. The subset of the 10 fittest animats with only one motor evolved even larger main complexes and also more concepts outside of the main complex.

As demonstrated above, restricting the sensor capacities of the animats increased brain integration since Task 1 had to be solved based on memory alone instead of momentary sensor states. Restricting the animats' motor capacities still allows using the sensor state S_1_S_2_ = 11 to distinguish blocks of size 3 from size 1. Nevertheless, with just one available motor, reliance on memory should increase, since movements have to be coordinated across several time steps. This, in turn, should lead to more concepts and higher integration. Fig. 7 shows the results of another 50 LODs evolved in Task 1 with one of the animats' motors disabled (set to 0 at every time step).

Overall, restricting the animats' motor capacities to one motor led to larger main complexes with more concepts and higher integration (*Φ^Max^*) (U_98_ = 806/824/741, Z = −3.156/−3.028/−3.618, p = 0.002/0.002/0.000 for #MC elements/#MC concepts/*Φ^Max^*). With one motor only, the maximal fitness achieved was 87.5% (112/118 correct trials) in one LOD; average final fitness was 78.8±0.7%. Task 1 with one motor could thus not be compared at the same level of fitness as Task 1 with two motors. Instead, a subset of the 10 fittest animats is plotted in dark green in Fig. 7, in addition to the average across all 50 LODs (light green). In this subset, also the number of modular concepts was significantly increased compared to the standard Task 1 (U_58_ = 107.5, Z = −2.857, p = 0.004). The maximal fitness reached with one motor and one hidden element was 71.8%. 24/50 animats evolved the same wiring diagram as shown in Fig. 4B, but with only one motor element. The fittest animat (112/128 correct trials) evolved an integrated main complex with at most 3 elements and <*Φ^Max^*>  = 0.38. Positive correlation with fitness was also higher given just one motor: the number of concepts and *Σφ^Max^* correlated significantly in 40/50 LODs and MC measures in 34/50 LODs.

**Figure 8:**
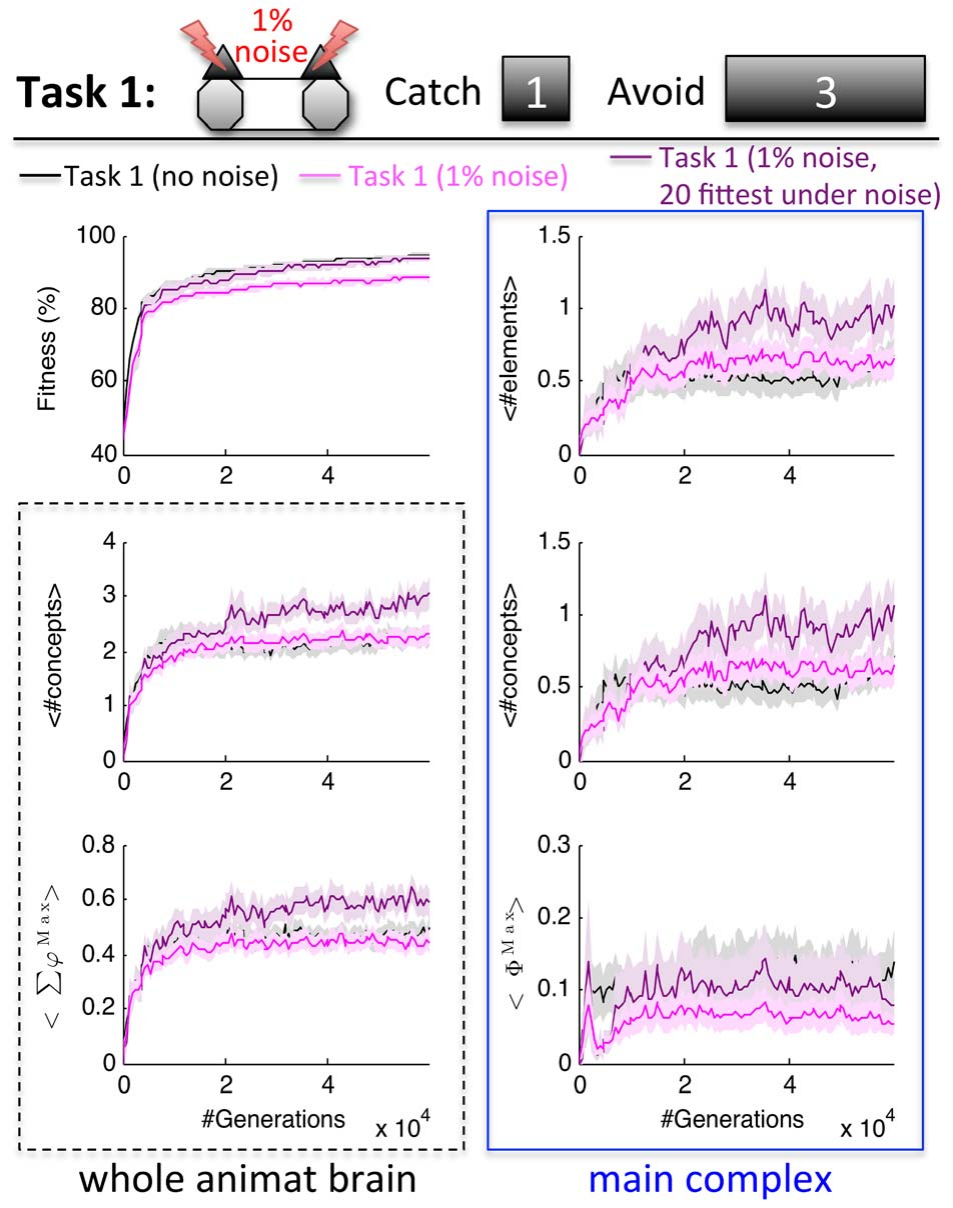
Concepts and integration in Task 1 with 1% sensor noise. The average fitness shown in the first plot is the percentage of correct trials in Task 1 tested in a noise-free environment. On average, adaptation with sensor noise decreased the animats' average fitness in the noise free environment of Task 1, without affecting the average number of concepts, <Σφ^Max^>, and the evolved main complexes. However, the subset of 20 LODs with the best final performance in the noisy environment (1% sensor noise, evaluated over 50 repetitions of each trial at generation 59,904) developed more concepts, <Σφ^Max^>, and larger main complexes with more MC concepts than those animats evolved in Task 1 without sensor noise, while reaching about the same level of fitness in the noise free condition.

Finally, evolutionary pressure for more memory should also arise with sensory data that are less reliable. Consequently, more concepts and higher integration are expected to evolve in an environment where sensor inputs are noisy, if compensating mechanisms are developed. To test this prediction, we simulated 50 additional LODs of Task 1 with 1% sensor noise for each of the two sensors (Fig. 8), meaning that the state of each sensor had a probability of 1% to be flipped. During evolution with noise, each trial was repeated 20 times and the next generation of animats was selected based on the average fitness across repetitions.

On average (Fig. 8, pink), animats evolved in the noisy environment developed brains with similar number of concepts and integration as those evolved in the noise-free environment (black). Presented with the noise-free Task 1, their average final fitness was lower than for those animats that had adapted to the noise-free environment (88.1±1.0% compared to 94.2±0.7%). Given the limited size of the animats' brains, it is possible that during 60,000 generations no compensatory mechanisms could be developed and the sensor noise only reduced the animats' performance without adaptive influence on their network structures. However, when fitness is evaluated in the environment *with* 1% sensor noise, the animats that had adapted to the noisy environment reached 79.0±0.8% fitness at generation 59,904, while the animats that had evolved without sensor noise only reached 76.3±0.7% fitness. This indicates that in a subset of the 50 evolutionary runs, the animats adapted to compensate for the sensor noise, at least in part. We thus evaluated the subset of 20 LODs evolved under noise with highest fitness in the noisy environment, shown in purple in Fig. 8. In line with the above predictions, this subset of LODs indeed showed more concepts and a trend for higher *Σφ^Max^*, and larger main complexes with more MC concepts than the animats that evolved without sensor noise (U_68_ = 299.0/368.0/380.0/382.0, Z = −2.638/−1.716/−1.658/−1.630, p = 0.008/0.086/0.097/0.103, respectively for #concepts/*Σφ^Max^/*#MC elements/#MC concepts), although their fitness in the noise-free Task 1 was very similar (first panel, Fig. 8). Note that, due to the data processing theorem [31], introducing sensor noise would generally decrease standard (Shannon) measures of information processing across the communication channel between the environment and the animat, regardless of compensatory mechanisms in the system. By contrast, measures of information integration may actually increase, since they take into account the noise compensation mechanisms implemented by the intrinsic causal structure of the animat. Taken together, the results presented in this section show that the number of concepts and their integration not only increase with the complexity of the environment, but also with the complexity of the environment relative to the sensor and motor capacities of the organism. This confirms the hypothesis that, if more reliance on memory is required to reach high levels of fitness and the number of elements is restricted, evolutionary pressure favors more integrated network structures.

## Discussion

In this study, we analyzed how the causal structure of simulated neural networks (animats) evolves during adaptation to environments of increasing complexity. To that end, we first evaluated all concepts (modular and integrated) specified by the brain elements of each animat and measured their integrated information *φ^Max^*. Second, we identified the animat's main complex (MC), the set of elements in an animat's brain that generates the maximally integrated conceptual structure, and computed its associated integrated conceptual information *Φ^Max^*.

We investigated the evolution of animats in four environments (Task 1–4) with different levels of task difficulty and requirements for sequential memory. Task difficulty (assumed to be inversely related to the average evolved fitness after 60,000 generations) was lowest for Tasks 1 and 2 and highest for Task 4. The requirements for sequential memory were low for Task 1, intermediate for Task 2 and 3, and high in Task 4. In accordance with the predictions of IIT, the animats evolved on average more concepts and larger, more integrated main complexes (higher *Φ*) the more sequential memory was necessary to solve a task. Similar results were obtained in a second set of simulations, in which the animats' sensor or motor capacities were restricted while the animats adapted to Task 1. This increased the reliance on memory and led, as predicted, to more concepts and more integrated conceptual structures. Taken together, these results point to an active evolutionary trend towards more concepts and integrated conceptual structures if the environment's causal structure is complex and there are constraints on the number of sensors, motors, and hidden elements.

### Informational measures of complexity

The notions of information and complexity play an important role in recent attempts to understand evolutionary success [2]–[4], [6], [7], [13], [20], [21], [32]. For example, Marstaller et al. [13] presented a measure of “representation”, defined in information-theoretic terms as the mutual information between (coarse-grained) states of the environment and internal “brain” states, given the states of the sensors. Applied to animats adapting to a block categorization task similar to Task 1, representation of a set of salient environmental variables was shown to increase during adaptation [13]. Another recent study examined how sensory-motor mutual information (*I_SMMI_*) [20], predictive information (*I_Pred_*) [21], and integrated information as defined in [6], change over the course of adaptation to a single environment with fixed statistical properties (traversing random mazes) [6], [7].

The mutual information between sensors and motors quantifies the degree of differentiation of the observed input-output behavior [20], [32]. Thus, *I_SMMI_* reflects the richness of a system's behavioral repertoire (behavioral complexity), which should be advantageous in a complex environment. Predictive information [21]—the mutual information between a system's past and future states—measures the differentiation of the observed internal states of a system. Thus, *I_Pred_* reflects the richness of a system's dynamical repertoire (dynamical complexity), which is also expected to promote adaptation to complex environments. *I_SMMI_*, *I_Pred_*, and integrated information as defined in [6] all increased during evolutionary adaptation to the maze environment [6], [7]. Moreover, these indices showed a positive correlation with fitness and positive lower bounds pointing to a minimal, necessary amount of complexity for a given fitness [7]. In the present simulations, *I_Pred_* always increased during evolution and was highest for Task 4 (see S4–S6 Figs.). However, changes in *I_SMMI_* with adaptation as measured in [6], [7], [13] varied with the task. Specifically, in Task 1 and 2, after an initial maximum *I_SMMI_* actually decreased with increasing memory capacity, as also observed in [13].

The present approach extends previous investigations in several ways. In addition to aggregate measures of information applied to the animat's brain as a whole, we evaluated all the individual concepts specified by the elements of each animat, taken alone or in various combinations (as specified in IIT 3.0 [15]). In essence, concepts characterize the irreducible input-output functions performed by a mechanism in a state [15]. Assessing concepts requires a perturbational approach that reveals a mechanism's causal properties within a system under all possible initial states [14], [15]. Thus, a concept expresses the entire set of causal dispositions or “powers” conferred by a mechanism in a given state to the system to which it belongs. This analysis thus picks up causes and effects, not just correlations, and does so for the entire set of possible circumstances to which an animat may be exposed, not just for those that happen to be observed in a given setting. Importantly, the causal analysis performed here also shows that combinations of elementary mechanisms (higher-order mechanisms) may specify additional concepts, thus greatly enriching the causal powers of an animat for a given number of elements. Crucially, higher-order concepts only count if they are integrated (*φ*>0), indicating that their causal power cannot be reduced to the causal power of their parts. For each animat in the present study the IIT 3.0 measures were evaluated for every brain state with p>0 and averaged, weighted by each state's probability of occurrence while the animat is performing the task. The finding that successful adaptation to more complex environments leads to the development of an increasing number of concepts fits well with the notion that, everything else being equal, different concepts provide different causal powers, thereby increasing the substrate available to selective processes.

The present results also show that complex environments lead not only to an increasing number of concepts available to an animat, but also to the formation of integrated conceptual structures within the animats' brains. If a conceptual structure specified by a set of elements is maximally irreducible to the conceptual structures specified by subsets of elements (*Φ^Max^*), the set of elements constitutes a main complex (MC) [15]. The conceptual structure specified by the main complex of an animat thus corresponds to a local maximum of causal power. In this way, the main complex forms a self-defined causal entity, whose borders are determined based on the causal powers of its own mechanisms. Importantly, while the concepts within a main complex are specified over hidden elements (the cause-effect repertoires are all within the MC), they do reflect previous input from the sensors and they can, of course, influence the motors. In this way, an integrated conceptual structure can combine current inputs and outputs with past ones and with the state of internal elements that may reflect past memories as well as future goals. All the concepts specified by the main complex over itself thus reflect a system's *intrinsic* complexity.

### When does evolution favor integrated structures?

Complexity and fitness are often associated, though not invariably [6], [7], [10], [33], [34]. In particular environmental niches, simple systems can be very successful, while complex systems may be selected against if, for example, increased energy requirements trump higher behavioral flexibility (e.g., [35]–[38]). For the evolution of intrinsic complexity investigated in this article, it is thus important to understand under which environmental conditions integrated conceptual structures become advantageous.

Overall, the results of the present simulations indicate that, given constraints on the number of elements and connections, integrated systems can have a selective advantage if the causal structure of the environment is complex. This was shown, first, by the finding that the highest fitness in the more complex tasks (2,3 and especially 4) was achieved by animats with (highly) integrated conceptual structures. By contrast, in a simpler task (Task 1), high fitness was achieved by both integrated and modular systems. Accordingly, correlations between measures of integration and fitness were low in Task 1, but increased progressively over Tasks 2–4 (Table 1, S1 Fig). The relative simplicity of Task 1 is illustrated by the rapid achievement of close to maximum fitness in most evolutionary histories and by the minimal requirement for sequential memory (in Tasks 2–4, a longer sequence of sensor inputs needs to be stored inside the animat's brain to perform adequately). Second, when Task 1 was made more difficult without changing the environment, by reducing the number of sensors and motors, animats had to rely more on sequential memory to achieve high fitness. In this case, animats that evolved highly integrated conceptual structures had once again a selective advantage.

Why is this so? Given limitations on the number of hidden elements, integrated brains can implement more functions (concepts) for the same number of elements, because they can make use of higher-order concepts, those specified by irreducible combinations of elements (see also [26]). Moreover, integrated brains with functions specified by hidden elements over hidden elements, or combinations of input, hidden, and output elements, are able to rely more on memory. Note that given an upper limit, or cost on the number of sensors, motors, and hidden elements (and the speed of interaction between them), an empirical positive lower bound of *Φ* will exist for higher fitness values in complex task environments, as observed for the informational measures evaluated in [7] (*I_SMMI_*, *I_Pred_*, and integrated information as defined in [16]). Note also, however, that any task could, in principle, be solved by a modular brain with *Φ = 0* given an arbitrary number of elements and time-steps (see in particular Fig. 21 in [15] and [39]–[41]).

Another potential advantage of integrated brains is related to degeneracy [25]. Degeneracy is the property according to which a given function can be performed by many different structures [25], [42], [43], and it is ubiquitous in biology [44]. Degenerate structures show equivalent behavior in certain contexts, but can perform different functions in different contexts. Degeneracy contrasts with redundancy, where many identical structures perform the same function under every circumstance. Systems that show high degeneracy usually are well-suited to integrating information [14], [25]. Indeed, our results are in line with higher degeneracy for animats having high *Φ*, both at the population level and within each individual animat brain. The number of different neural architectures, logic functions, and behaviors developed by animats with integrated brains (*Φ*>0) that solved Task 1 and 2 was much higher than for animats with modular brains (*Φ* = 0). More potential solutions with *Φ*>0 provide a probabilistic selective advantage for integrated structures and lead to higher variability due to neutral mutations (S3 Fig.) and more heterogeneous populations. This suggests that populations having high *Φ* and high degeneracy should be better at adapting rapidly to unpredictable changes in the environment and more robust to mutations, because some animats are likely to be available that are already predisposed to solve new problems.

A similar advantage is provided by degeneracy in the concepts available to each individual animat. In integrated brains, selective pressure may favor the emergence of particular concepts. However, in such brains higher order concepts will also become available at no extra cost in terms of elements or wiring, and they may prove useful to respond to novel events. How the evolution of integrated conceptual structures with high degeneracy is affected by changing environments, or by environments with multiple connected niches and coevolution of different species [45] will be the subject of future work.

To conclude, rich environments that put a premium on context-sensitivity and memory, such as competitive social situations, should favor the evolution of organisms controlled by brains containing complexes of high *Φ*. This is because the integrated conceptual structures specified by complexes of high *Φ* can accommodate a large number of functions in a way that is more economical and flexible than what can be achieved with modular or nearly-modular architectures. Moreover, since according to IIT integrated conceptual structures underlie consciousness [14], [15], [18], [23], the finding that such structures offer a selective advantage in complex environments could provide a rationale as to why and how consciousness evolved.

## Methods

### Animats

Animat brains consist of 8 binary elements: 2 sensors, 4 hidden elements, and 2 motors (left, right) that can loosely be referred to as neurons. The sensors are directed upwards with a space of one unit between them and activated (set to 1) if a falling block is located directly above a sensor (Fig. 1). Otherwise the sensor element is set to 0. All elements are updated from time step *t* to *t+1* according to a transition probability matrix (TPM). In general, the TPM could be probabilistic with transition probabilities between 0 and 1. In the present work, however, the animats' TPMs are purely deterministic, i.e., transition probabilities are either 0 or 1. The brain elements can thus be considered as binary Markov variables, whose value is specified by deterministic logic gates (just as the Markov brains in [13]). Note that the elements are not limited to classic logic gates, such as ANDs, ORs, or XORs, but can potentially specify any deterministic logic function over their inputs. If only one of the motors is updated to state 1, in the next time step the animat will move one unit to the right (motor state 01) or left (motor state 10), respectively. Since no other movement was required of the animat, motor state 11 (both motors on) was chosen to be redundant with motor state 00, for which the animat will not move.

To evaluate the number of different TPMs and connectivity matrices for animats with perfect fitness in Task 1 and 2, the TPMs and connectivity matrices were compared in “normal form”, i.e., independent of the labels of their elements and only potentially causal connections were included in the analysis (meaning, hidden elements with only inputs or outputs to the rest of the system were excluded). To that end, for a given matrix all elements were permuted and the resulting permuted matrices were ordered lexicographically. The first permuted matrix was then chosen as the “normal form”.

All animat brains are initialized without connections between their elements. Connectivity evolves indirectly during adaption to the environment as outlined below, following a genetic algorithm that selects, mutates, and updates the animat's genome at each new generation. The animats' genes encode hidden Markov gates (HMGs), which in turn determine the connectivity and transition table of each brain element: each HMG has input elements, output elements, and a logic table that specifies the elements' transition table (see [6], [13] for details). In this study, the ancestral genome (generation 0) of all animats does not encode any HGM. Different from previous publications [6], [7], [13], evolution is thus not “jump-started”, which avoids random causal connections in the animats' brains, but requires more generations to reach high levels of fitness.

The animats' genomes consist of at least 1,000 and at most 20,000 loci, where each locus in the genome is an integer value ∈ [0,255]. The beginning of a gene is marked by a start codon (the consecutive loci 42 and 213), followed by two loci that respectively encode the number of inputs and outputs of one HMG. The next eight loci are used to determine where the inputs come from and the outputs go to. Because gates are allowed to have at most 4 in- and at most 4 outgoing connections, 8 loci are reserved, and used according to the 2 preceding loci. The subsequent loci encode the transition table of the HMG, determining the input and output elements and their logical relations. This encoding is robust in the sense that mutations that change the input-output structure of an HMG only add or remove the respective parts of the HMG's logic table, while the rest of the table is left intact. Encoding the connectivity and logic functions of the animats' brain elements with HMGs allows for recurrent connections between hidden elements and also self-connections. Feedback from the hidden elements to the sensors, and also from the motors to the hidden units is however prohibited by zeroing out the sensors and motors at each time-step respectively before the new sensor input arrives and after the movement was performed.

### Environment

The animat is located at the bottom row of a 16×36 unit world with periodic boundary conditions (Fig. 2B). We chose the height of 36 units to allow the animats enough time to assess the direction and size of the falling blocks from each initial condition. Each animat is tested in 128 trials: all 16 initial block positions, with blocks moving to the right and left, and four potentially different block sizes. Note that in Task 1 (“catch size 1, avoid size 3”) and Task 2 (“catch size 1, avoid size 2”) the two different block sizes are thus shown 2×32 = 64 times, while in Task 3 (“catch size 1+4, avoid size 2+3”) and Task 4 (“catch size 3+6, avoid size 4+5”) each block size is shown 32 times. In each trial a block of a certain size falls from top to bottom in 36 time steps, moving 1 unit downwards and sideways always in the same direction (left or right). If at time-step 36 at least one of the animat's units overlaps with the block, it is counted as “caught”, otherwise as “avoided”.

In Task 1, sensor state S_1_S_2_ = 11 unambiguously distinguishes size 3 blocks from size 1 blocks. In all other cases, whether a block should be caught or avoided cannot be decided based on a momentary sensor input state.

### Fitness and Genetic Algorithm

An animat's fitness *F* at each generation is simply calculated as the percentage of successfully caught and avoided blocks out of all possible 128 test trials. Starting from a set of 100 ancestral animats without HMGs and thus without connections between elements, the animats adapt according to a genetic algorithm across 60,000 generations. At each generation, fitness is assessed for all animats in a population of 100 candidates. The most successful candidates are selected probabilistically for differential replication according to an exponential fitness measure *S* = 1.02^F*128^. For every successfully caught or avoided block the score is thus multiplied by 1.02. The 100 candidate animats are ranked according to *S* and selected into the next generation with a probability proportional to *S* and thus to their fitness (roulette wheel selection without elite). After this replication step, the new candidate pool is mutated in three different ways: a) by point mutations, which occur with a probability of p = 0.5% per locus, causing the value to be replaced by a random integer drawn uniformly from [0,…,255]; b) by deletion: with 2% probability, a sequence between 16 and 512 adjacent loci is deleted; c) by duplication: with 5% probability a sequence between 16 and 512 adjacent loci is duplicated and inserted at a random location within the animat's genome, where the size of the sequence to be deleted or duplicated is uniformly distributed in the range given. Since insertions are more likely than deletions, genomes tend to grow in size during evolution. Deletions and duplications are, however, constrained so that the genome remains between 1,000 and 20,000 loci. All genes are expressed. Some of the genes may give rise to redundant HMGs, which, however, will not be robust to mutation. Under fitness selection, the number of genes thus tends to converge to a balanced level (roughly the number of possible elements). Under random selection, only very few rapidly changing random connections between elements appear, and existing network structures decompose within less than 1,000 generations [7].

For each task, 50 evolutionary runs of 60,000 generations are performed. At the end of each evolutionary run, the line of descent (LOD) [19] of a randomly chosen animat from the final generation is traced back to its initial ancestor at generation 0. For each evolutionary run one LOD is obtained, which captures the run's particular evolutionary history. Since reproduction is asexual, without crossover, a unique LOD can be identified for an animat from the final generation. Because, moreover, all animats are part of the same niche, it makes almost no difference which animat is chosen in the final generation, since going backwards across generations their different LODs quickly coalesce to a single line [6]. We performed the full IIT analysis across each line of descent every 512 generations starting from 0.

### IIT analysis

**Figure 9:**
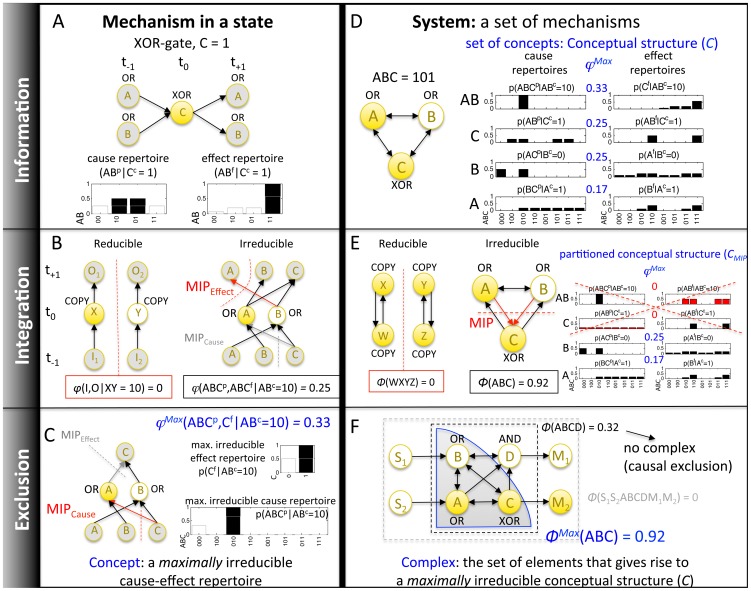
The information, integration, and exclusion postulate applied at the level of mechanisms (A–C) and systems of mechanisms (D–F). (A–F) Each node is a binary logic-gate mechanism that can be in either state ‘0’ (white) or ‘1’ (yellow). The logic-gates and their connections are represented as neural circuits rather than electronic circuits: directed connections between the nodes indicate the inputs and outputs of the logic gates. The mechanisms labeled A, B, and C correspond to system ABC = 101 shown in (D). (A) Information: Mechanism C in its current state ‘1’ generates information as it constrains its causes (the past states of its inputs AB) and effects (the future states of its outputs AB) compared to their unconstrained distributions (gray distribution). Past and future nodes whose state is unspecified are shown in gray. (B) Integration: The elements X and Y do not form an integrated higher order mechanism, since XY is reducible to its component mechanisms X and Y (φ = 0). However, the elements AB in state ‘10’ do form a higher order mechanism, since AB specifies both, irreducible causes and irreducible effects (the minimum information partition (MIP) on both, the cause and effect side leads to a loss of information). Integrated information φ of AB = 10 is evaluated as the minimum of the cause and effect integrated information: φ =  min(φ_Cause_, φ_Effect_), here φ =  φ_Effect_  = 0.25, taking all inputs and outputs of AB into account. The overall MIP of AB over all its inputs and outputs is thus MIP_Effect_, labeled in red. (C) Exclusion: Of all input-output combinations of mechanism AB, the “concept” of AB = 10 is its maximally irreducible cause repertoire, here over all input elements ABC (φ_Cause_ = 0.33, same as in (B)), together with its maximally irreducible effect repertoire, here over output element C only (φ_Effect_ = 0.5). This means that AB has its maximally irreducible effect repertoire specified on C, not on ABC or any other output combination. The concept's integrated information is φ^Max^  =  min(φ_Cause_, φ_Effect_)  =  φ_Cause_  = 0.33, its overall MIP is MIP_Cause_, labeled in red. (D) System information: The system ABC = 101 gives rise to a conceptual structure with 4 concepts. (E) System integration: The system WXYZ is reducible into the subsets WX and YZ. WXYZ cannot exist as a system from the intrinsic perspective. By contrast, system ABC is irreducible. Its minimum information partition (MIP) leaves the concepts of A and B intact, but destroys concepts C and AB. Integrated conceptual information Φ(ABC) is evaluated as the difference between the whole conceptual structure C and the partitioned conceptual structure C_MIP_ (see Text S2 in [15]). (F) System exclusion: Of all sets of elements in this larger system, the set ABC has Φ^Max^ and thus forms the main “complex”. ABCD, for example, also specifies integrated conceptual information Φ, but cannot form another complex since it overlaps with ABC and Φ(ABC)> Φ(ABCD) (see Fig. 2).

The most recent mathematical formulation of the integrated information theory (“IIT 3.0”) is presented in detail in [15]. In the following we will summarize the main principles and measures relevant to this study, illustrated in simple examples of neuron-like logic gates mechanisms (Fig. 9).

#### Mechanisms and concepts

From the intrinsic perspective of a system, a mechanism has a causal role within the system (a “difference that makes a difference”) if its present state constrains the potential past and future states of the system compared to the unconstrained distribution (the distribution of past and future states if all input states to each element are equally likely). This is assessed by perturbing the system into all possible states and observing the effects on the system [25], [46], yielding a transition probability matrix (TPM) that contains the probability of transitioning from each system state to every other system state. As a simple example, Fig. 9A shows mechanism C in state ‘1’. C is a XOR logic gate that receives inputs from elements A and B and outputs again to A and B. The full system ABC is displayed in Fig. 9D. The fact that C is an XOR gate and that at present it is in state C = 1 inherently constrains the past state of the system (“cause repertoire” 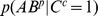, i.e., only AB = [10, 01] are possible causes) as well as the future state (“effect repertoire” 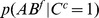, i.e., AB = [11] is the only possible effect), as compared to the unconstrained distribution (indicated in gray). The superscripts p, c, f label “past”, “current”, and “future” system subsets. The same approach can be used to evaluate the cause-effect repertoire of higher order mechanism (combinations of elements), such as AB, which is used in what follows to illustrate the notion of integration. In sum, the cause- and effect-repertoire of a mechanism are conditional probability distributions over sets of system elements, albeit not using observed distributions (as done for correlational measures), but considering all system states with equal probability.

From the intrinsic perspective, only integrated information matters: the whole has to specify a cause-effect repertoire that is not reducible to that of its parts (Fig. 9B). Irreducibility is assessed by causally partitioning subsets of elements by introducing noise into the connections between them [14], [15]. The partitioned cause-effect repertoire then corresponds to the product distribution of the cause-effect repertoires specified by the parts. If a mechanism can be partitioned without loss of information, as in the case of XY in Fig. 9B (left), the combined mechanism XY cannot have a causal role above and beyond the causal roles of X and Y separately. By contrast, the elements A and B of the example system ABC do form a 2^nd^ order mechanism AB (Fig. 9B, right). This is because AB constrains the past *and* future of the system ABC more than A and B separately. The amount of integrated (irreducible) information a mechanism *M* specifies in its current state *s_0_* is quantified by *φ*, which measures the distance between the whole and partitioned cause-effect repertoire. The partition used to evaluate *φ* is the minimum information partition (MIP), the partition that makes the least difference. *φ* is determined on both the cause and the effect-side: 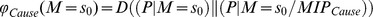
and 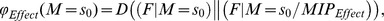
where *P* and *F* denote a set of system elements in the past and future, respectively. Differences *D* between distributions are assessed via the earth-mover's distance (EMD). Generally, EMD quantifies the minimal cost of transforming one probability distribution into another specified over a “ground distance” between system states [15], [47]–[49]. Contrary to the commonly used Kullback-Leibler divergence [31], the EMD is a metric. That is, it is symmetric, bounded, and takes the distance between individual system states into account, here measured by their Hamming distance. The state ‘110’, for example, is more distant from ‘001’ (Hamming distance of 3) than from ‘100’ (Hamming distance of 1). Transporting p = 0.25 from state ‘110’ to ‘001’ would thus corresponds to an EMD of 0.25*3 = 0.75, while transporting it to ‘100’ corresponds to an EMD of 0.25.

From the intrinsic perspective of the system, the amount of integrated information specified by a mechanism in a state cannot be more than either the cause or the effect integrated information, so the minimum of the two is taken [15]:
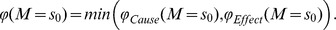



Finally, again from the intrinsic perspective, the mechanism's causal role within the system in its current state can only be a single one—corresponding to the cause-effect repertoire that is *maximally* irreducible (a mechanism cannot perform multiple input-output functions over an overlapping set of elements, [15]). Thus, *φ* must be calculated for all possible input and output combinations of the mechanism. For the example mechanism AB (Fig. 9C), the cause repertoire of AB = 10 over all past elements ABC is the maximally irreducible cause repertoire, with 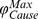  = 0.33 (transporting p = 0.33 from state ‘010’ to state ‘000’). On the effect side, the effect repertoire of AB = 10 over future element C is the maximally irreducible one with 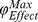  = 0.5 (transporting p = 0.5 from state ‘1’ to state ‘0’; compare to Fig. 9B: the effect repertoire of AB = 10 over all elements ABC only has 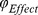  = 0.25). The maximally irreducible cause and effect repertoire with *φ^Max^*(AB = 10)  =  min(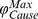,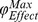) defines the mechanism's “concept”, the core causal role of the mechanism in its current state from the intrinsic perspective of the system itself. Following a principle of causal exclusion, all other inputs and outputs of the mechanism are treated as unconstrained.

#### System of mechanisms and main complex

At the system level, the set of all concepts specified by a system of mechanisms in its current state constitutes a conceptual structure (Fig. 9D). For example, the system ABC = 101 specifies a conceptual structure comprising 4 concepts: 3 elementary, or 1^st^ order concepts of its elementary mechanisms A, B, and C, and the 2^nd^ order concept AB.

As for a mechanism and its causal role, a system of mechanisms forms a “causal entity” from its own intrinsic perspective only if the conceptual structure it specifies cannot be reduced to that specified by its parts. Specifically, each part of the system must have both causes and effects in the other part (“strong integration”, Fig. 9E, middle), otherwise some elements could never influence the system or be influenced by it. Irreducibility at the level of systems of mechanisms is quantified by partitioning the system elements unidirectionally. This means that the inputs or outputs of a subset of elements are rendered causally ineffective by noise. Integrated conceptual information *Φ* (“big phi”) measures the difference between the conceptual structure *C* of the whole system *S* in state *s_0_* and the conceptual structure *C_MIP_* of the partitioned system:



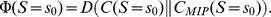



The difference *D* between two conceptual structures is evaluated using an extended version of the earth-mover's distance (EMD), which quantifies the minimal cost of transforming the conceptual structure *C* of the whole to the conceptual structure *C_MIP_* of the partitioned set of elements. Instead of probabilities, in the *extended* EMD it is the *φ* values of the concepts that are redistributed from conceptual structure *C* to *C_MIP_*. Instead of the Hamming distance, the “ground distance” between the concepts of *C* and *C_MIP_* is given by the EMD distance of their cause-effect repertoires. Since *Σφ^Max^* of all concepts of *C* is usually higher than that of *C_MIP_*, any residual *φ^Max^* is transported to the “null” concept (the unconstrained distribution). For more details and an explicit example see Text S2 in [15].

Within a system, many sets of elements can potentially give rise to integrated conceptual structures. However, from the intrinsic perspective of a system, there can only be a single conceptual structure over a set of elements, with no overlap with other conceptual structures, barring a multiplication of causes and effects (causal exclusion). Once again, the relevant conceptual structure is the one that is maximally irreducible (*Φ^Max^*), and the corresponding set of elements constitutes a “complex” – a self-defined causal entity within the system. The complex with maximal *Φ* in the system is called the “main complex” (MC). Note that, in principle, *Φ^Max^* should be evaluated over the spatio-temporal scale at which causal interactions are strongest [50]. Since an animat's MC is comprised of maximally 4 hidden Markov elements, we assume these micro elements to be the relevant spatio-temporal scale.

In the system shown in Fig. 9F, ABC forms the main complex (see also Fig. 2D). Note that, if the subset of a system is analyzed, such as ABC in Fig. 9F, the remaining elements act as background conditions (fixed external constraints). The number of elements, the number of concepts, and the *Φ* value of a main complex are measures of integration in a system (Fig. 9F: 3 MC elements, 4 MC concepts, and *Φ^Max^* = 0.92). Note that a complex always consists of at least 2 elements, since a single element, even if it has memory in form of a self-loop, cannot be partitioned. Moreover, feed-forward structures cannot give rise to a complex and have *Φ* = 0. For the same reason the whole system S_1_S_2_ ABCD M_1_M_2_, as well as every subsystem that includes a sensor or motor element is not integrated and has *Φ* = 0.

Modular mechanisms (feed-forward chains, self-loops, and mechanisms outside the main complex) can of course also contribute to the evolutionary success (fitness) of an organism. The number of concepts in the whole system, here S_1_S_2_ ABCD M_1_M_2_, provides a measure of all causal relations in the system, modular and integrated, and the sum of their φ^Max^ values is a measure of their combined strength (see Fig. 3B: 6 concepts: A, B, C, D, AB, AC, Σφ^Max^ = 1.08).

### Statistics

Table 1 shows the average (nonparametric) Spearman rank correlation coefficients across all 50 LODs for all evaluated IIT measures in Task 1-4. In S1 Fig. complementary histograms are shown of the correlation coefficients of all individual LODs. Correlation coefficients were calculated based on ranked variables (i.e., using Spearman's instead of Pearson's correlation coefficients), since the amount by which fitness increases is not expected to depend linearly on any of the causal measures. Initial increases in fitness can be large, simply because initially there is more room for large improvements than at later generations where the animat already has a high percentage of fitness.

Error margins throughout this article denote SEM.

Since none of the measured variables was found to be normally distributed for all task conditions (Kolmogorov-Smirnoff test for normality) and variances between tasks differed for some of the measures, statistical differences were evaluated using a Kruskal-Wallis test, the non-parametric equivalent of a one-way ANOVA. For all statistical tests across task conditions after adaptation, measures were averaged over the last 3,000 generations (6 data points).

Task 1–4 were compared (see Fig. 3), first, taking all 50 independent LODs of each task into account, despite the lower average fitness reached in Task 3 and 4. In this set, statistical differences were found for the number of concepts, *Σφ^Max^*, and *Φ^Max^* (p = 0.001/0.002/0.016), but not for the number of MC concepts and MC elements. Second, Task 1–4 were compared at the same level of fitness, taking only a subset of LODs with high final fitness into account in Task 3 and 4 (9 and 7 fittest LODs, respectively). The respective subsets of LODs were selected as the set of fittest LODs in Task 3 and 4, whose average fitness across the last 5,000 generations was closest to that achieved on average in Task 1. Compared at the same level of fitness, all IIT measures showed statistical differences (p = 0.000/0.000/0.003/0.003/0.000 for #concepts/*Σφ^Max^*/#MC elements/#MC concepts/*Φ^Max^*).

Moreover, the standard Task 1 was compared to Task 1 with one sensor only, one motor only, and 1% sensor noise (Fig. 6–8). All measures showed significant difference (p = 0.000) when all 50 LODs of each condition were taken into account and also when a subset of LODs with high fitness was compared (again, p = 0.000 for all measures).

Differences between pairs of task conditions reported in the results section were assessed by post-hoc Mann-Whitney U tests.

Custom-made MATLAB software was used for all calculations. The program to calculate the complex of a small system of logic gates and its constellation of concepts is available under [51]. EMD calculations within the IIT program were performed using the open source fast MATLAB code of Pele and Werman [49]. The IBM SPSS software package was used for statistical analysis.

## Supporting Information

S1 Fig **Distribution of correlation coefficients with fitness in the four different Task conditions for all 50 individual LODs.** For each LOD, Spearman's correlation coefficient between fitness and each of the displayed causal measures was calculated across the evolved 60,000 generations. In the histograms, shaded bars denote number of significant correlations with fitness (positive and negative). White bars show additional numbers of non-significant correlation coefficients. Blue lines indicate the overall average of correlation coefficients <R> listed in Table 1 (main text). For all measures, the number of LODs that correlated positively with fitness increased from Task 1 to Task 4. In all tasks, the number of concepts in the whole animat brain and their *Σφ^Max^* values showed higher correlation with fitness.
(PDF)

S2 Fig **Dissociations between causal measures of IIT.** For each Task 1-4, an individual example LOD is shown, in which a dissociation between the different IIT measures can be observed (indicated by the red vertical lines). In the LOD of Task 1, at the indicated jump in fitness, *Φ^Max^* decreases, while all other measures increase. In the example LOD of Task 2, the jump in fitness is accompanied by an increase in the overall number of concepts and their *Σφ^Max^*, while the MC measures decrease. Note that initially, for low fitness, the animat brains tend to first develop modular concepts that are not integrated (*Φ^Max^* = 0, see also Fig. 4A, main text). In the 3^rd^ LOD (Task 3), *Σφ^Max^* and *Φ^Max^* both increase during the indicated rise in fitness, while the number of overall concepts stays constant and the average number of MC concepts and MC elements decreases. In the 4^th^ LOD (Task 4) the overall number of concepts and their *Σφ^Max^* increase with fitness, while the MC concepts and MC elements decrease, and *Φ^Max^* stays constant. Since in the animats the maximum number of MC elements is 4, the number of MC concepts here is closely linked to the number of MC elements. Nevertheless, in general, for larger systems, the number of MC concepts can far exceed the number of MC elements (2^N^-1, where N is the number of MC elements) and will thus be much more variable for a fixed number of MC elements.
(PDF)

S3 Fig **Variability of TPMs across generations for two representative Task 1 example LODs in which the animats evolve perfect fitness with a modular or integrated brain structure.** The upper panels show the evolution of fitness in the two LODs with *Φ^Max^* = 0 (left) and *Φ^Max^*>0 (right) at the end of the evolutionary run. The lower panels show the Hamming distance of the animats' TPMs between consecutive generations as a measure for the variability of the causal structure of the animats' brains during adaptation. The Hamming distance counts the number of TPM entries (0s or 1s) in which two TPMs differ from each other. Note that for this purpose, the TPMs were permuted into a normal form that allows comparing the causal structure independent of the element label. This means that if the causal structure stays the same, but e.g. two hidden elements switch their causal roles the measured Hamming distance between them is still 0. With *Φ^Max^* = 0 the LOD's TPMs do not vary much once perfect fitness is reached. When perfect fitness is maintained with *Φ^Max^*>0, however, the LOD's TPMs still vary considerably between consecutive generations. This can be explained by the higher degeneracy of animats with perfect fitness and *Φ^Max^*>0 (see main text), which allows for neutral mutations in the population and also more heterogeneous populations with the same probability of being selected into the next generation.
(PDF)

S4 Fig **Evolution of sensory-motor mutual information and predictive information in Task 1-4.** The sensory-motor mutual information (*I_SMMI_*) was evaluated between the distribution of sensor states at t_0_ and the distribution of motor states at t_+1_. The predictive information (*I_Pred_*) corresponds to the mutual information between brain states at t_0_ and t_+1_, including sensors, hidden elements, and motors. Calculating the two measures across 2 time-steps, i.e., between t_0_ and t_+2_, results in qualitatively similar results with somewhat lower values (data not shown). The terminology used here corresponds to that in [7]; different from [6], [13] where *I_SMMI_* was termed predictive information, while *I_Pred_* was termed *I_total_* in [6]. As observed in [13], *I_SMMI_* is initially high in Task 1 and decreases with adaptation. This is because, initially, direct connections between sensors and motors can increase fitness in Task 1. Once memory is evolved, however, *I_SMMI_* decreases. The drop in *I_SMMI_* from generation ∼0 to 20,000 thus indicates that the motors become less dependent on sensor inputs and are driven more by the hidden elements. In Task 4 direct connections between sensors and motors alone cannot increase fitness, and thus are not evolved in the early generations, which leads to an increase in I_SMMI_ from low values to the level observed in Task 1. Task 2 and 3 are intermediate in this respect. I_SMMI_ is bounded both by the entropy of the sensors (*H_Sen_*) and the entropy of the motors (H_Mot_). H_Sen_ depends mostly on the respective task (size of blocks), as can be seen from the different initial values across Task 1–4. In Task 2 *H*_Sen_ is particularly low, because sensor state S_1_S_2_ = 11 is impossible. Interestingly, H_Mot_ increases during adaptation and reaches the same level in all tasks for the same level of fitness. I_Pred_ quantifies the amount of information that the current system state contains about the next state of the system. Note that the animats' brains are comprised of deterministic Markov elements. I_Pred_ should thus be maximal across one time-step. I_Pred_ is bounded by the entropy of system states (H_State_) and follows it closely. While I_Pred_ and H_State_ are the same for Task 1 and 2, the higher values for Task 3 and 4 can be explained by the higher number of hidden elements, and thus higher potential entropy, evolved in the more difficult tasks. In summary, neither I_SMMI_, nor I_Pred_ capture the increasing complexity of Task 1–4 for the animat in terms of requirements for sequential memory, but rather depend on external task characteristics (entropy of sensor input) and the number of evolved hidden elements.
(PDF)

S5 Fig **Sensory-motor mutual information and predictive information for two Task 1 example LODs in which the animats evolve brains with a modular or integrated structure.** *I_SMMI_*, *I_Pred_*, and the sensor, motor, and state entropy are displayed for two Task-1 example LODs that reach perfect fitness (compare Fig. 4, main text). For details on the measures see S4 Fig. and [7]. The sensory-motor mutual information *I_SMMI_* decreases with fitness in both LODs, following the decrease in entropy of the sensor inputs (*H_Sen_*). The predictive information (*I_Pred_*) evolves to similar values in the LOD that evolves a modular structure (Fig. 4B) and the one that evolves an integrated structure (Fig. 4C). *IPred* thereby follows *H_State_*, which also reaches similar values in both cases. Note that in both LODs the animats evolved brains with 2 hidden elements, which can partly explain why they show similar values of *H_State_*.
(PDF)

S6 Fig **Evolution of sensory-motor mutual information and predictive information in Task 1 with sensor or motor restrictions.** For details on the measures see S4 Fig. and [7]. While for only one functional sensor and noisy sensor inputs the sensory-motor mutual information I_SMMI_ does not differ much from standard Task1, I_SMMI_ for only one functioning motor is greatly reduced. I_SMMI_ thus seems to depend more on the entropy of the motor units, H_Mot_. The predictive information (I_Pred_) evolves to similar values, regardless of sensor or motor constraints, as does the entropy of system states (H_State_), which can be explained by similar evolved number of elements: the lower entropy due to a missing sensor or motor is compensated by more hidden elements. Neither I_SMMI_, nor I_Pred_ detect increases in the intrinsic complexity of the animats due to sensor or motor constraints.
(PDF)

S1 Text **Theoretical upper bounds for *Σφ^Max^* and *Φ^Max^*.**
(DOC)
