# Supplementary figures and images for: Evolution of Integrated Causal Structures in Animats Exposed to Environments of Increasing Complexity
Source: PLoS Comput Biol. 2014 Dec 18;10(12):e1003966. doi: 10.1371/journal.pcbi.1003966 (PMC4270440; doi:10.1371/journal.pcbi.1003966)

# Task 1

# Task 2

# Task 3

# Task 4

Catch

1

1

1

4

3

6

Avoid

3

2

2

3

4

5

whole animal brain

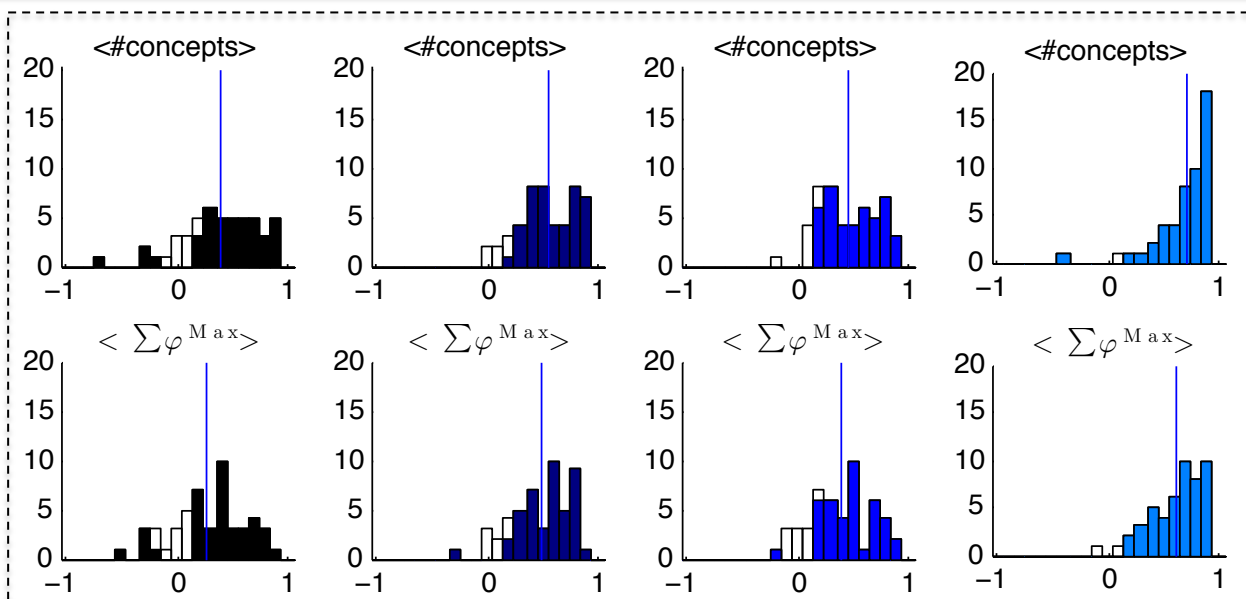

main complex

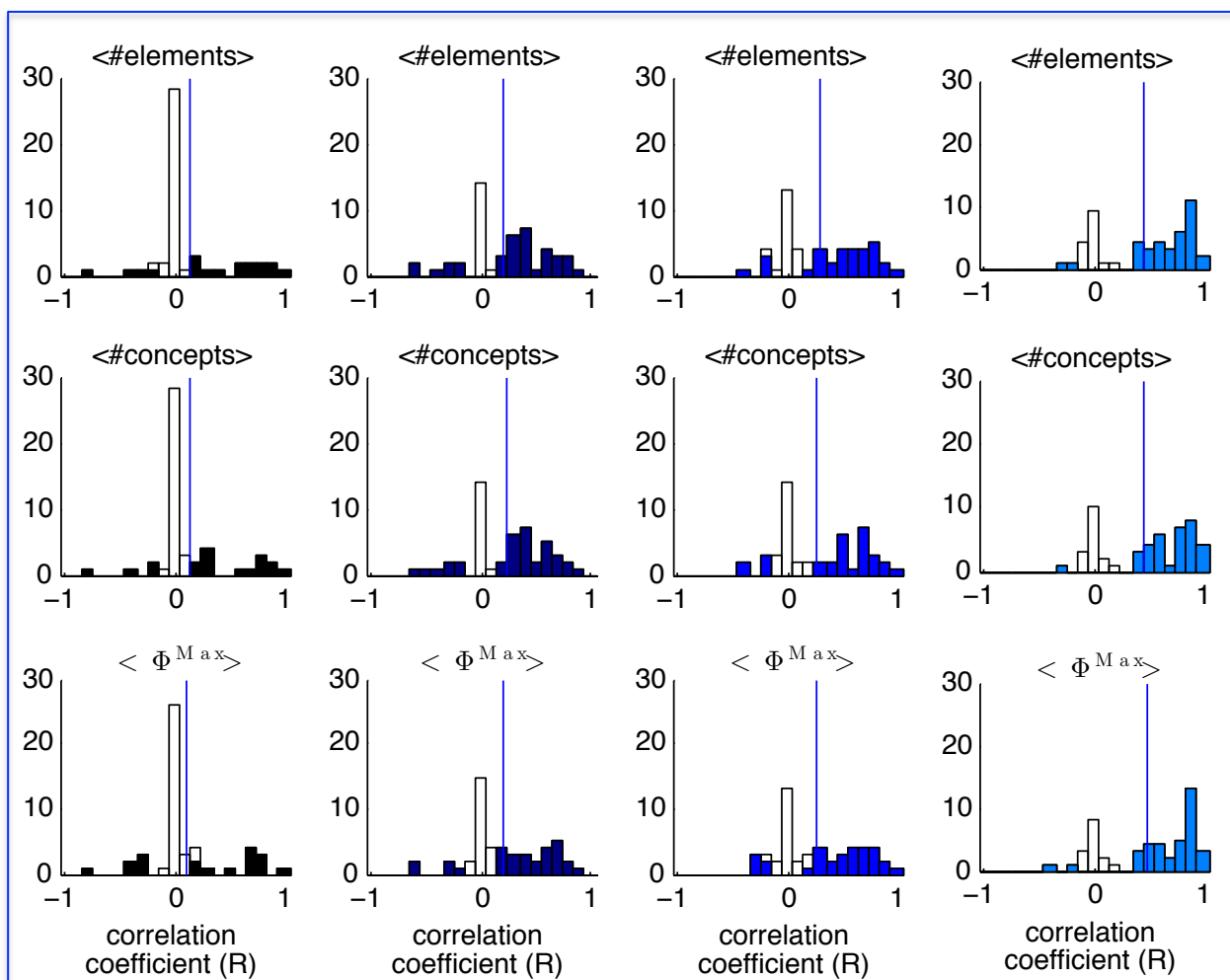

Supplement: S1 Fig — Distribution of correlation coefficients with fitness in the four different Task conditions for all 50 individual LODs. For each LOD, Spearman's correlation coefficient between fitness and each of the displayed causal measures was calculated across the evolved 60,000 generations. In the histograms, shaded bars denote number of significant correlations with fitness (positive and negative). White bars show additional numbers of non-significant correlation coefficients. Blue lines indicate the overall average of correlation coefficients listed in Table 1 (main text). For all measures, the number of LODs that correlated positively with fitness increased from Task 1 to Task 4. In all tasks, the number of concepts in the whole animat brain and their ΣφMax values showed higher correlation with fitness. (PDF) [file pcbi.1003966.s001.pdf]

Task 1

Task 2

Task 3

Task 4

whole animal brain

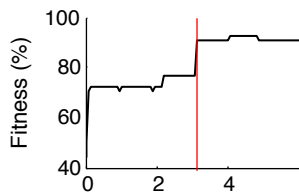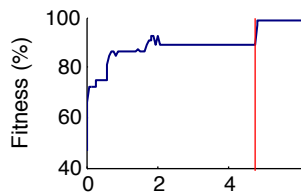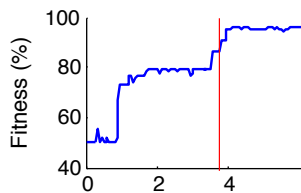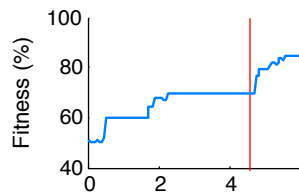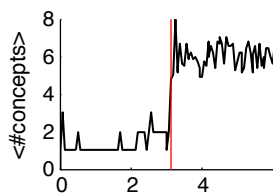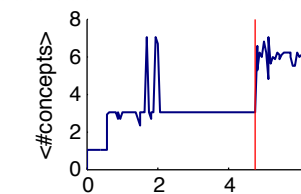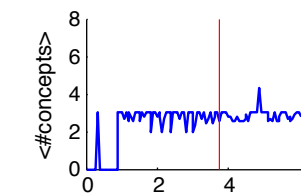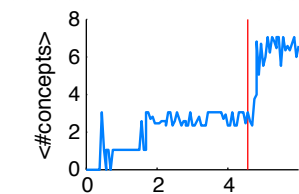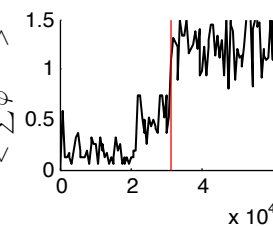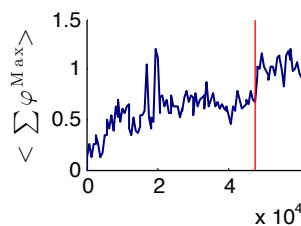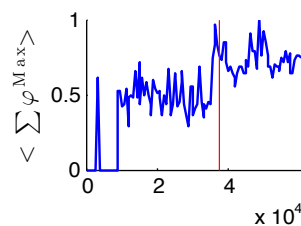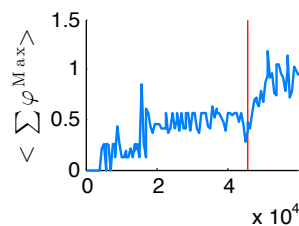

main complex

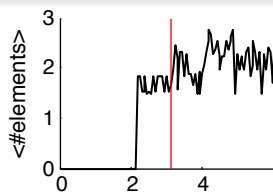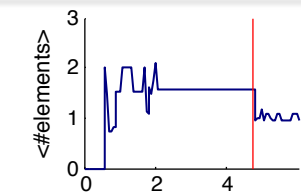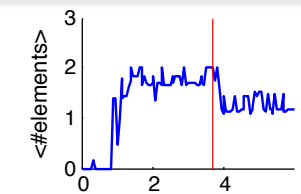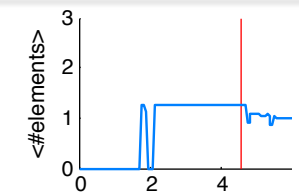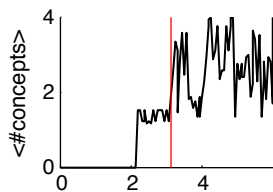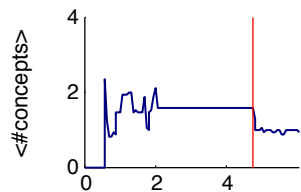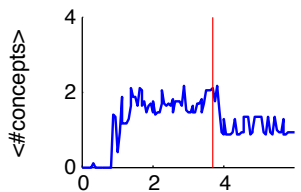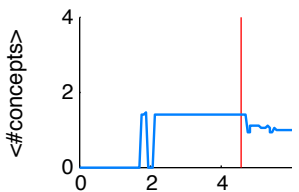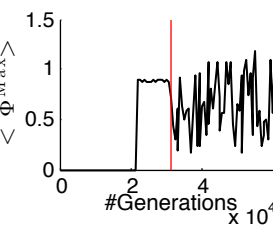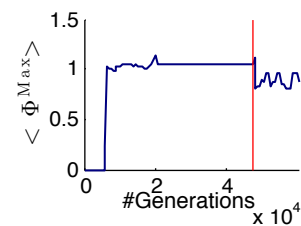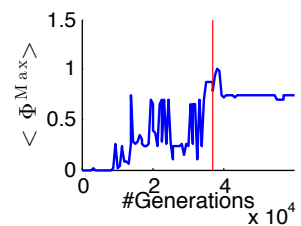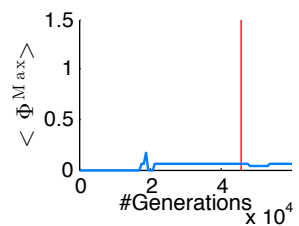

Supplement: S2 Fig — Dissociations between causal measures of IIT. For each Task 1-4, an individual example LOD is shown, in which a dissociation between the different IIT measures can be observed (indicated by the red vertical lines). In the LOD of Task 1, at the indicated jump in fitness, ΦMax decreases, while all other measures increase. In the example LOD of Task 2, the jump in fitness is accompanied by an increase in the overall number of concepts and their ΣφMax, while the MC measures decrease. Note that initially, for low fitness, the animat brains tend to first develop modular concepts that are not integrated (ΦMax = 0, see also Fig. 4A, main text). In the 3rd LOD (Task 3), ΣφMax and ΦMax both increase during the indicated rise in fitness, while the number of overall concepts stays constant and the average number of MC concepts and MC elements decreases. In the 4th LOD (Task 4) the overall number of concepts and their ΣφMax increase with fitness, while the MC concepts and MC elements decrease, and ΦMax stays constant. Since in the animats the maximum number of MC elements is 4, the number of MC concepts here is closely linked to the number of MC elements. Nevertheless, in general, for larger systems, the number of MC concepts can far exceed the number of MC elements (2N-1, where N is the number of MC elements) and will thus be much more variable for a fixed number of MC elements. (PDF) [file pcbi.1003966.s002.pdf]

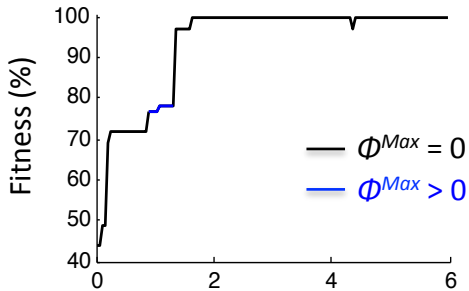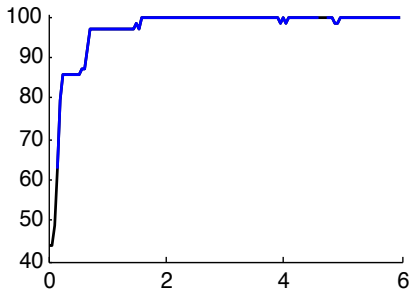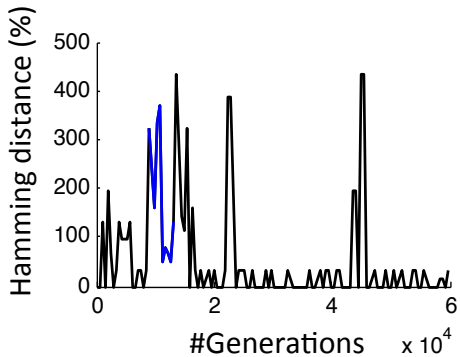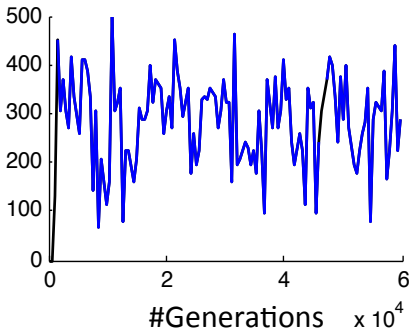

Supplement: S3 Fig — Variability of TPMs across generations for two representative Task 1 example LODs in which the animats evolve perfect fitness with a modular or integrated brain structure. The upper panels show the evolution of fitness in the two LODs with ΦMax = 0 (left) and ΦMax>0 (right) at the end of the evolutionary run. The lower panels show the Hamming distance of the animats' TPMs between consecutive generations as a measure for the variability of the causal structure of the animats' brains during adaptation. The Hamming distance counts the number of TPM entries (0s or 1s) in which two TPMs differ from each other. Note that for this purpose, the TPMs were permuted into a normal form that allows comparing the causal structure independent of the element label. This means that if the causal structure stays the same, but e.g. two hidden elements switch their causal roles the measured Hamming distance between them is still 0. With ΦMax = 0 the LOD's TPMs do not vary much once perfect fitness is reached. When perfect fitness is maintained with ΦMax>0, however, the LOD's TPMs still vary considerably between consecutive generations. This can be explained by the higher degeneracy of animats with perfect fitness and ΦMax>0 (see main text), which allows for neutral mutations in the population and also more heterogeneous populations with the same probability of being selected into the next generation. (PDF) [file pcbi.1003966.s003.pdf]

## Task 1

Catch **1**  
Avoid **3**

## Task 2

**1**  
**2**

## Task 3

**1** **4**  
**2** **3**

## Task 4

**3** **6**  
**4** **5**

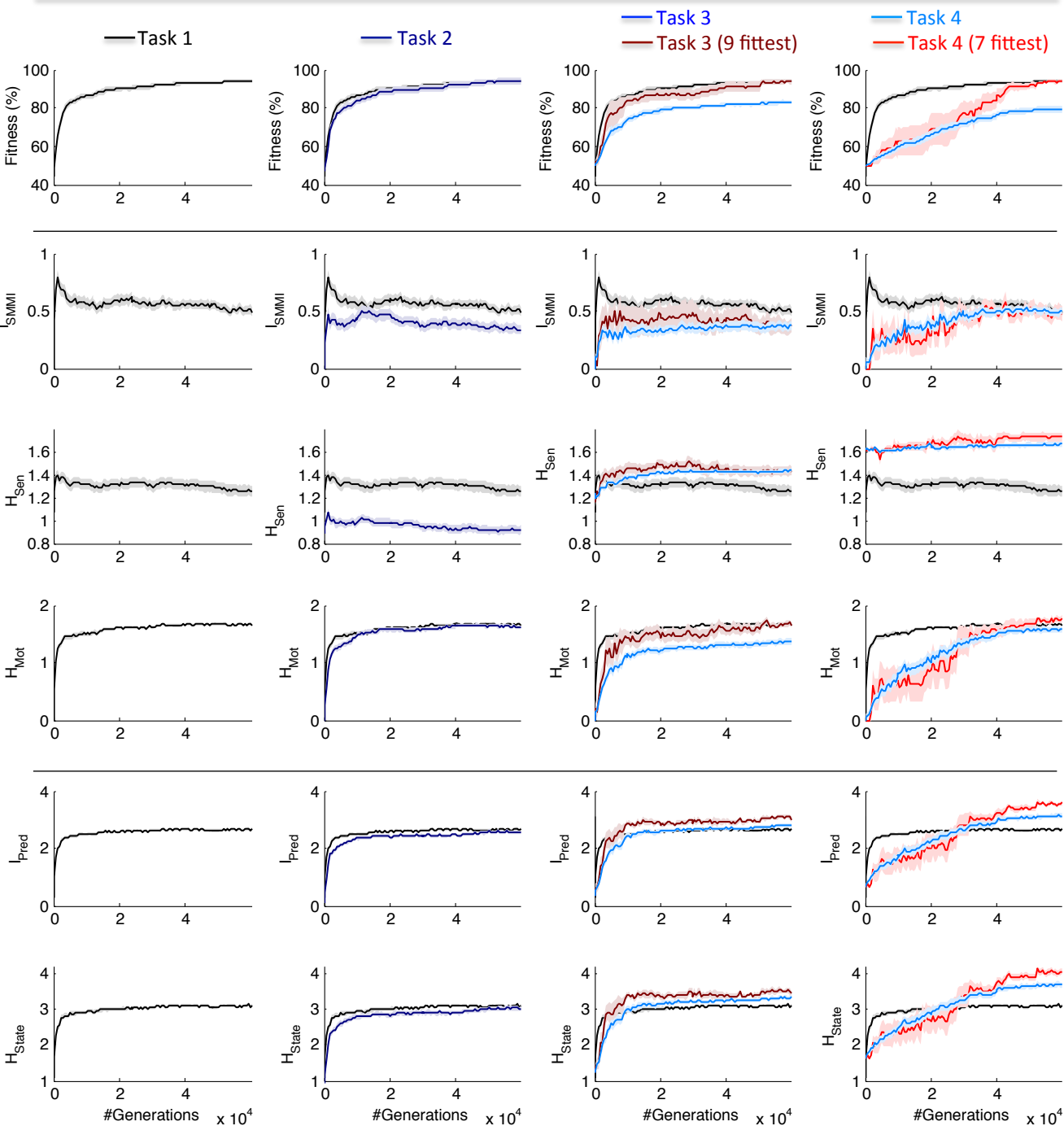

Supplement: S4 Fig — Evolution of sensory-motor mutual information and predictive information in Task 1-4. The sensory-motor mutual information (ISMMI) was evaluated between the distribution of sensor states at t0 and the distribution of motor states at t+1. The predictive information (IPred) corresponds to the mutual information between brain states at t0 and t+1, including sensors, hidden elements, and motors. Calculating the two measures across 2 time-steps, i.e., between t0 and t+2, results in qualitatively similar results with somewhat lower values (data not shown). The terminology used here corresponds to that in [7]; different from [6], [13] where ISMMI was termed predictive information, while IPred was termed Itotal in [6]. As observed in [13], ISMMI is initially high in Task 1 and decreases with adaptation. This is because, initially, direct connections between sensors and motors can increase fitness in Task 1. Once memory is evolved, however, ISMMI decreases. The drop in ISMMI from generation ∼0 to 20,000 thus indicates that the motors become less dependent on sensor inputs and are driven more by the hidden elements. In Task 4 direct connections between sensors and motors alone cannot increase fitness, and thus are not evolved in the early generations, which leads to an increase in ISMMI from low values to the level observed in Task 1. Task 2 and 3 are intermediate in this respect. ISMMI is bounded both by the entropy of the sensors (HSen) and the entropy of the motors (HMot). HSen depends mostly on the respective task (size of blocks), as can be seen from the different initial values across Task 1–4. In Task 2 H Sen is particularly low, because sensor state S1S2 = 11 is impossible. Interestingly, HMot increases during adaptation and reaches the same level in all tasks for the same level of fitness. IPred quantifies the amount of information that the current system state contains about the next state of the system. Note that the animats' brains are comprised of deterministic M [file pcbi.1003966.s004.pdf]

# Task 1:

Catch

1

Avoid

3

— Task 1 — example of modular LOD — example of integrated LOD

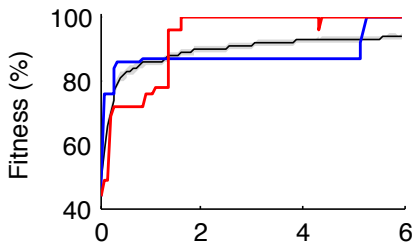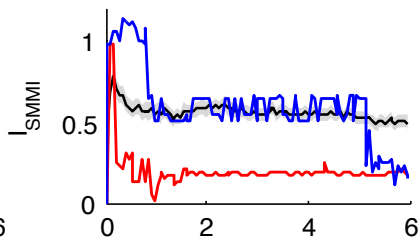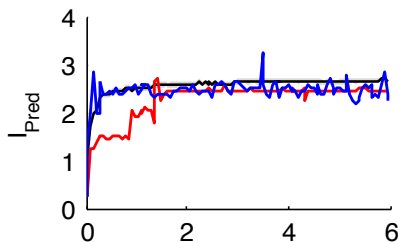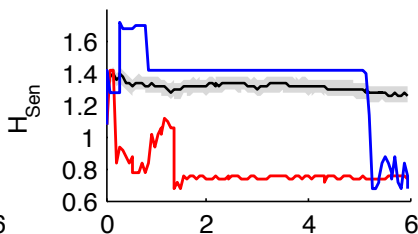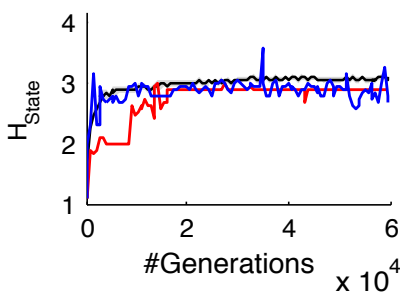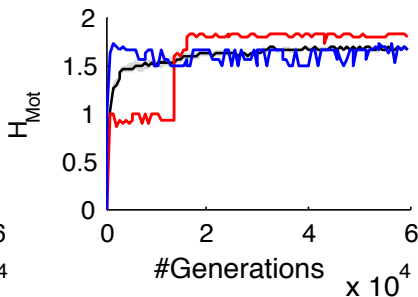

Supplement: S5 Fig — Sensory-motor mutual information and predictive information for two Task 1 example LODs in which the animats evolve brains with a modular or integrated structure. ISMMI, IPred, and the sensor, motor, and state entropy are displayed for two Task-1 example LODs that reach perfect fitness (compare Fig. 4, main text). For details on the measures see S4 Fig. and [7]. The sensory-motor mutual information ISMMI decreases with fitness in both LODs, following the decrease in entropy of the sensor inputs (HSen). The predictive information (IPred) evolves to similar values in the LOD that evolves a modular structure (Fig. 4B) and the one that evolves an integrated structure (Fig. 4C). IPred thereby follows HState, which also reaches similar values in both cases. Note that in both LODs the animats evolved brains with 2 hidden elements, which can partly explain why they show similar values of HState. (PDF) [file pcbi.1003966.s005.pdf]
