# Supplementary material for: Evolution of Integrated Causal Structures in Animats Exposed to Environments of Increasing Complexity
Source: PLoS Comput Biol. 2014 Dec 18;10(12):e1003966. doi: 10.1371/journal.pcbi.1003966 (PMC4270440; doi:10.1371/journal.pcbi.1003966)
Supplement: S6 Fig — Evolution of sensory-motor mutual information and predictive information in Task 1 with sensor or motor restrictions. For details on the measures see S4 Fig. and [7]. While for only one functional sensor and noisy sensor inputs the sensory-motor mutual information ISMMI does not differ much from standard Task1, ISMMI for only one functioning motor is greatly reduced. ISMMI thus seems to depend more on the entropy of the motor units, HMot. The predictive information (IPred) evolves to similar values, regardless of sensor or motor constraints, as does the entropy of system states (HState), which can be explained by similar evolved number of elements: the lower entropy due to a missing sensor or motor is compensated by more hidden elements. Neither ISMMI, nor IPred detect increases in the intrinsic complexity of the animats due to sensor or motor constraints. (PDF) [file pcbi.1003966.s006.pdf]

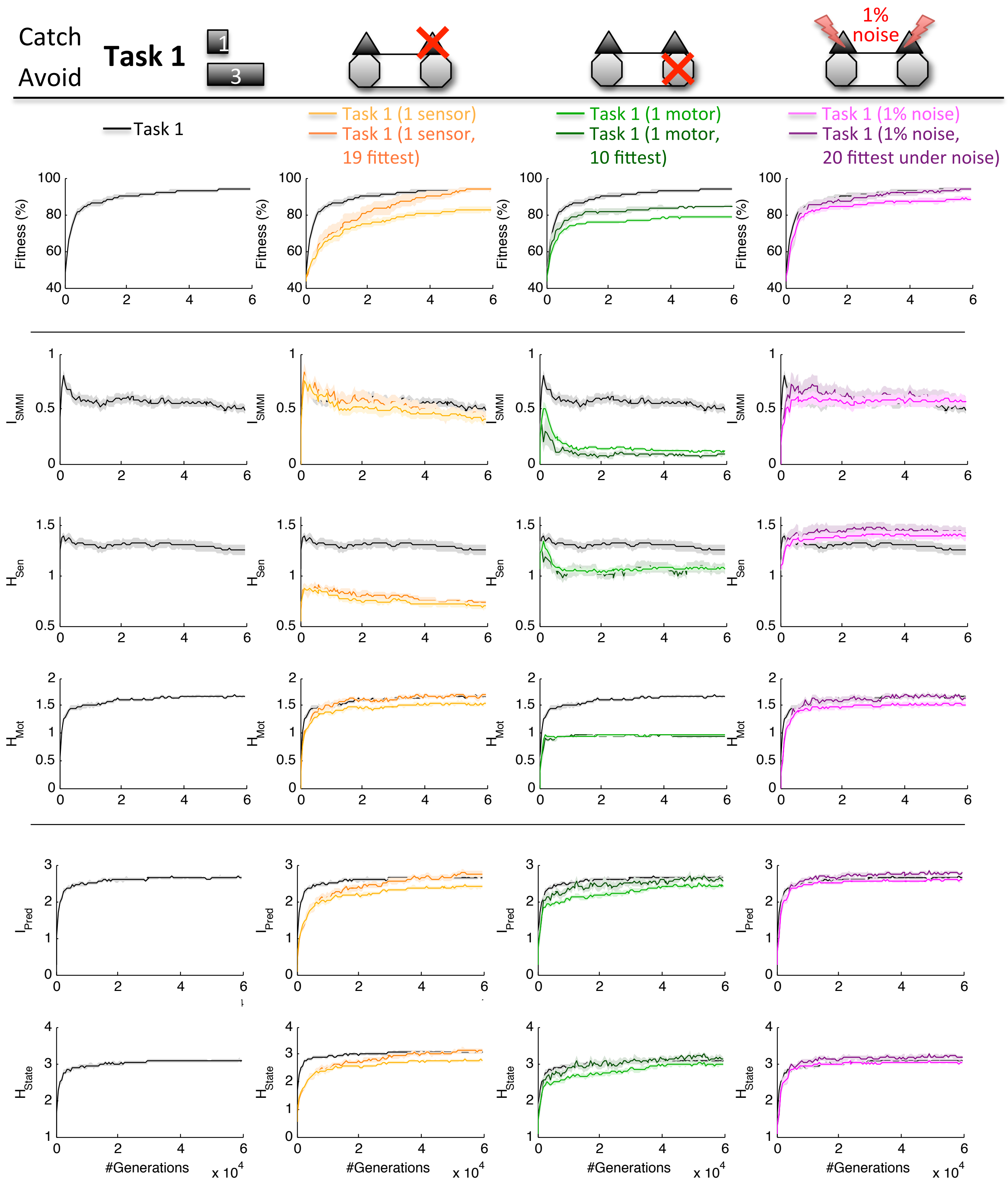

Catch
Avoid
Task 1
1
3
1% noise
Task 1
Task 1 (1 sensor)
Task 1 (1 sensor, 19 fittest)
Task 1 (1 motor)
Task 1 (1 motor, 10 fittest)
Task 1 (1% noise)
Task 1 (1% noise, 20 fittest under noise)
Fitness (%)
I_SMMI
H_Sen
H_Mot
I_Pred
H_State
#Generations
x 10^4
