# Supplementary material for: Evolution of Integrated Causal Structures in Animats Exposed to Environments of Increasing Complexity
Source: PLoS Comput Biol. 2014 Dec 18;10(12):e1003966. doi: 10.1371/journal.pcbi.1003966 (PMC4270440; doi:10.1371/journal.pcbi.1003966)
Supplement: S1 Text — Theoretical upper bounds for ΣφMax and ΦMax. (DOC) [file pcbi.1003966.s007.doc]

Text S1 - Theoretical upper bounds for *Σϕ^Max^* and *Φ^Max^.*

For a particular number of elements, upper bounds must exist for both, *Σϕ^Max^* and *Φ^Max^*, since the earth mover’s distance (EMD) for probability distributions is bounded. Given N binary elements, the maximal EMD between two probability distributions is N (e.g. transporting a probability p = 1 from state “all OFF” to state “all ON”). Note that *ϕ^Max^* and *Φ^Max^* measure irreducible (intrinsic) information, corresponding to the notion of “differences that make a difference”, not to be confused with Shannon information (see Text S3 in [1]). Being based on the Hamming distance between system states, *ϕ^Max^* and *Φ^Max^­* are pure numbers and not measured in bits.

For *Σϕ^Max^*, the animats’ cause and effect repertoires are probability distributions about at most 6 elements each: the 4 hidden elements and 2 sensors in case of the cause repertoire, the 4 hidden elements and 2 motors in case of the effect repertoire. Each animat brain can at most have 15 concepts (power-set of the 4 hidden elements, excluding the empty set, i.e., 2^4^-1 = 15). If we assume that the maximal possible value of *ϕ^Max^* of each concept is the maximal possible EMD distance to any distribution, i.e., *ϕ^Max^*=6, *Σϕ^Max^* is at most 15*6 = 90. Note, however, that the assumption of *ϕ^Max^*=6 for all concepts is impossible to achieve in practice, since higher order concepts could not exist if elementary concepts completely specify the system’s past and future state. Moreover, typical *ϕ^Max^* values are considerably lower than N. In practice, without interaction between elements (as e.g. for the inputs of an XOR gate) *ϕ^Max^* cannot be higher than 0.5, equivalent to losing information about one input or output node. The theoretical upper limit of *Σϕ^Max^* is thus expected to be closer to 15*0.5 = 7.5.

Similarly, assuming that i) the MC comprises all 4 hidden elements, ii) all 15 concepts exist, iii) *ϕ^Max^* = 4 for every concept (the maximal possible EMD given that MC concepts can be about the 4 MC elements only), and iv) all concepts are maximally altered by the minimum information partition (MIP), the absolute upper bound for *Φ^Max^* in an animat brain is *Φ^Max^* = 15*4*8 = 480, where 8 is the maximal distance between the cause-effect repertoire of a concept in the whole and the partitioned constellation (see Text S2). Again, it is in fact impossible for a system to fulfill all the above assumptions and the expected theoretical bound of *Φ^Max^* is considerably lower. Under the same considerations as above, a more reasonable upper bound is expected to be on the order of *Φ^Max^*=15*0.5*(0.5+0.5) = 7.5, assuming all 15 concepts are lost by the partition. Deriving more stringent theoretical upper bounds on *Σϕ^Max^* and *Φ^Max^* is beyond the scope of this paper and will be the subject of future work. In line with the order of magnitude of the practically approximated upper bounds, the overall highest observed values for an animat in a particular state were *Σϕ^Max^*= 3.11 and *Φ^Max^* = 4.125.

1. Oizumi M, Albantakis L, Tononi G (2014) From the Phenomenology to the Mechanisms of Consciousness: Integrated Information Theory 3.0. PLoS Comput Biol 10: e1003588. doi:10.1371/journal.pcbi.1003588.
